# ISRES+: an improved evolutionary strategy for function minimization to estimate the free parameters of systems biology models

**DOI:** 10.1093/bioinformatics/btad403

**Published:** 2023-06-24

**Authors:** Prasad Bandodkar, Razeen Shaikh, Gregory T Reeves

**Affiliations:** Department of Chemical Engineering, Texas A&M University, 3122 TAMU, College Station, TX 77843, United States; Department of Chemical Engineering, Texas A&M University, 3122 TAMU, College Station, TX 77843, United States; Department of Chemical Engineering, Texas A&M University, 3122 TAMU, College Station, TX 77843, United States; Interdisciplinary Program in Genetics and Genomics, Texas A&M University, College Station, TX 77843, United States

## Abstract

**Motivation:**

Mathematical models in systems biology help generate hypotheses, guide experimental design, and infer the dynamics of gene regulatory networks. These models are characterized by phenomenological or mechanistic parameters, which are typically hard to measure. Therefore, efficient parameter estimation is central to model development. Global optimization techniques, such as evolutionary algorithms (EAs), are applied to estimate model parameters by inverse modeling, i.e. calibrating models by minimizing a function that evaluates a measure of the error between model predictions and experimental data. EAs estimate model parameters “fittest individuals” by generating a large population of individuals using strategies like recombination and mutation over multiple “generations.” Typically, only a few individuals from each generation are used to create new individuals in the next generation. Improved Evolutionary Strategy by Stochastic Ranking (ISRES), proposed by Runnarson and Yao, is one such EA that is widely used in systems biology to estimate parameters. ISRES uses information at most from a pair of individuals in any generation to create a new population to minimize the error. In this article, we propose an efficient evolutionary strategy, ISRES+, which builds on ISRES by combining information from all individuals across the population and across all generations to develop a better understanding of the fitness landscape.

**Results:**

ISRES+ uses the additional information generated by the algorithm during evolution to approximate the local neighborhood around the best-fit individual using linear least squares fits in one and two dimensions, enabling efficient parameter estimation. ISRES+ outperforms ISRES and results in fitter individuals with a tighter distribution over multiple runs, such that a typical run of ISRES+ estimates parameters with a higher goodness-of-fit compared with ISRES.

**Availability and implementation:**

Algorithm and implementation: Github—https://github.com/gtreeves/isres-plus-bandodkar-2022.

## 1 Introduction

In systems biology, information from large volumes of sequencing and spatiotemporal gene expression data is typically condensed to build mathematical models that can describe the data concisely and make predictions to guide experimental design. These models tend to have several phenomenological (statistical) or mechanistic parameters. Statistical model parameters determine correlation among experimental data while mechanistic model parameters have biophysical interpretations such as binding/dissociation rates of protein complexes. Biological systems have complex dynamics, making it usually difficult to experimentally measure all the parameters. Inverse modeling is applied to estimate parameters by minimizing an objective function that measures the error between model predictions and experimental data. Such a parameter estimation problem can be represented as a function optimization problem where the objective is to estimate the values of the parameters that reduce a measure of error between model predictions and experimental data (Hengenius 2014).

These models are usually expressed in the form of coupled ordinary or partial differential equations, where the state variables are protein/mRNA concentrations which are then fit to the experimental data (Hengenius 2014). From a geometric perspective, these models form a hyper-surface of all possible predictions in high-dimensional data space called the model manifold. The structure of the manifold makes it difficult to correctly estimate certain parameters (Ashyraliyev *et al.* 2009a, Mannakee *et al.* 2016). A global optimization approach to estimate these parameters is to employ Nature-inspired algorithms (NIAs) such as evolutionary strategies, simulated annealing, ant colony optimization, and particle swarm optimization to solve complex problems with scant data and a range of feasible solutions in multidimensional hyper-planes using metaheuristic techniques (Kar 2016, Kumar *et al.* 2023). Genetic algorithms (GAs) are an evolutionary strategy (ES) inspired by biological evolution. They evaluate the fitness of randomly generated solutions and apply mechanisms such as reproduction, natural selection, mutation, and recombination to generate a set of potential solutions and attempt to converge on a globally optimum solutions (Sun *et al.* 2012). Particularly, the Stochastic Ranking Evolutionary Strategy (SRES) uses stochastic ranking to avoid local minima (Carrell *et al.* 2017). It enables faster convergence and a higher number of feasible solutions (Moles *et al.* 2003, Mezura-Montes and Lopez-Ramirez 2007). SRES is widely used in systems biology to estimate model parameters due to superior performance compared with other optimization algorithms (Moles *et al.* 2003, Fomekong-Nanfack *et al.* 2007, Fakhouri *et al.* 2010, Liberman *et al.* 2017, Parmar and Mendes 2019, Rrapaj *et al.* 2021). An Improved Stochastic Ranking Evolutionary Strategy (ISRES) uses a penalty function to constrain feasible solutions and has been shown to perform better than SRES (Runarsson and Yao 2005). SRES and ISRES have been implemented to solve multiobjective optimization problems in systems biology (Maeda *et al.* 2019). This has enabled means to reverse-engineer signaling pathways (Ashyraliyev *et al.* 2009b, Jostins and Jaeger 2010, Crombach *et al.* 2012, Kozlov *et al.* 2012), infer the dynamics of signaling networks (Lobo and Levin 2015, O’Connell and Reeves 2015, Liu *et al.* 2016, Filkova *et al.* 2019), and understand the design principles of gene regulatory networks (Spirov and Holloway 2013).

If a vector of model parameters represents an individual in a population, the ISRES (Runarsson and Yao 2005) algorithm is basically a strategy for the evolution of a population of randomly chosen individuals in search space to a population that is tightly clustered around the fittest individual. The fittest individual at the end of the evolution would represent the final vector of model parameters that best fit the model predictions to the data. In a constrained optimization problem, the fitness of an individual is determined by evaluating an objective function that measures the error between model predictions and data, and a penalty function determines the feasibility of the solution. ISRES generates an initial population of random individuals from a uniform distribution. All the individuals in a generation are ranked based on a fitness score that is assigned to them by evaluating the objective and penalty functions. ISRES uses stochastic ranking to balance the objective and penalty. A fixed number of highest ranked individuals are selected to produce offspring to create a new population. This is done partially by recombination, where the fittest individual mates with the other selected individuals, and partially by mutation of the constituent parameters of the selected individuals. Thus, the recombination component biases the search while the mutation component ensures all individuals contribute to the search. In this manner, the algorithm converges to a region around a function minimum and continues to improve its estimate by exploring successively smaller regions of the search space by creating tightly distributed population clusters.

In this article, we present ISRES+, an upgraded algorithm that builds on the ISRES (Runarsson and Yao 2005). ISRES+ takes advantage of the excess information generated by the ES. In every generation, a population of a large number of individuals is created to explore the fitness landscape but only a few are selected to generate progeny for the next generation. Our goal is to not only use information from the population but also from the lineage of all individuals in the population to better understand the fitness landscape. We use linear least squares to fit either a linear or a quadratic model to the data in every generation. The linear least squares fit coefficients can then be correlated to the coefficients of a Taylor series expansion to construct an approximate gradient and an approximate Hessian. A linear model is used to build an estimate of the gradient, which is used to perform an approximate gradient descent step and a quadratic function model is used to build an estimate of the Hessian, which is used to perform an approximate Newton’s method of function optimization step. In every generation, in addition to the recombination and mutation contributions, new individuals are created from top-ranked individuals by the linear least squared fits and added to the population. Note that these methods add only minimal overhead to the creation of new offspring since neither the gradient nor the Hessian is really computed, such that ISRES+ takes a similar wall-clock time compared with ISRES (Supplementary Section S5). Both are estimated using the coefficients of the linear least squares fit. To maintain maximum progress, the fitting is done in the local neighborhood of the fittest individual found in all generations. The population size is kept constant in every generation by adding these individuals at the expense of some of the mutation contributions of the lowest-ranked individuals. The aim is to provide a better estimate of the search direction that complements the recombination method of the ES and improve the efficiency of ISRES such that a typical run of ISRES+ will more likely converge to a better solution than ISRES.

We test the performance of ISRES+ in estimating model parameters for three widely applied systems biology models and found that when the linear least squares fit-based methods contribute ∼2% to a new population in every generation, there was a higher probability of converging to a better solution for the same set of hyperparameters that characterize the ES, such as the population size and the number of generations. In addition, the final distribution of best parameter values was narrower with the addition of the linear least squares fit contributions to ISRES compared with without it. Finding a better fit parameter set allows the models to be better calibrated to make predictions and infer underlying regulatory mechanisms.

## 2 Methodology

In this study, we test the performance of the ISRES+ algorithm on three systems biology models described by Ordinary Differential Equations (ODEs) and compare it with ISRES (Runarsson and Yao 2005). The choice of these models is motivated by numerous factors such as model complexity, the number of free parameters, the number of state variables, and the number of spatiotemporal points (Table 1). The first model, referred to as the Dorsal/Cactus (Dl/Cact) model, describes a morphogen system that patterns the dorsal–ventral axis of early *Drosophila* embryos (Kanodia *et al.* 2009, O’Connell and Reeves 2015, Schloop *et al.* 2020). The second model, referred to as the Smad signaling model, describes TGF-β-induced Smad2 signaling in HaCat cells (Schmierer and Hill 2007, Schmierer *et al.* 2008). The third model, referred to as the gap gene circuit model, describes the patterning of the anterior–posterior axis of early *Drosophila* embryos (Jaeger *et al.* 2004, Manu *et al.* 2009, Surkova *et al.* 2009) (see Supplementary Section S1 for additional details on the systems biology models).

**Table 1.** Model complexity across systems biology models investigated in the study.

Model	Number of free parameters	Number of optimization points	Number of system variables
Model 1: Dl/Cact model	15	∼12 000	∼300
Model 2: Smad signaling model	10	∼50	25
Model 3: Gap gene circuit model	44	∼1800	228

The objective function is defined as the sum of squares error according to the following equation: where x is the parameter vector and s and t are the spatial and time coordinates. When applicable, constraints on the feasible regions are expressed as, where m is the number of constraints. The fitness function is then represented as, where the penalty function $\phi \left(x\right)$ is a quadratic penalty function defined as and r is a vector of penalty coefficients that regulates the dominance between the objective and the penalty functions in determining fitness. ISRES uses stochastic ranking to handle constraints in a way that neither under-penalizes (low r) nor over-penalizes (high r) the fitness function. The aim of stochastic ranking is to maintain a balance between the objective and penalty functions ensuring that an optimum search direction is maintained not just in the overall search space but in the feasible search space (Runarsson 2002). We use the fitness function suggested by the authors for ISRES for all three models (see Supplementary Section S1).


(1) $$f\left(x\right)=\sum_{s,t}{\left[y{\left(x,s,t\right)}_{\mathrm{model}}\;-\;y{\left(s,t\right)}_{\exp}\;\right]}^{2},$$

(2) $$g_{j}\left(x\right)\leq 0,\;\mathrm{where}\;j=1,2,..\;m,$$

(3) $$\psi \left(x\right)=f\left(x\right)+r\phi \left(g_{j}\left(x\right)\right),$$

(4) $$\phi \left(g_{j}\left(x\right)\right)=\sum_{j=1}^{m}\max{\left(0,g_{j}\left(x\right)\right)}^{2}$$

The search boundaries for the parameters of each of the models ranges from 1*e* − 4 to 1*e* + 4, unless it is known from experimental observations or scaling arguments that a parameter is restricted to vary in a different range (see Supplementary Section S1). The objective is to find a value for the parameter vector x, that the algorithm would characterize as the fittest individual found over the course of evolution across all generations.

## 3 Algorithm

**Figure 1.**
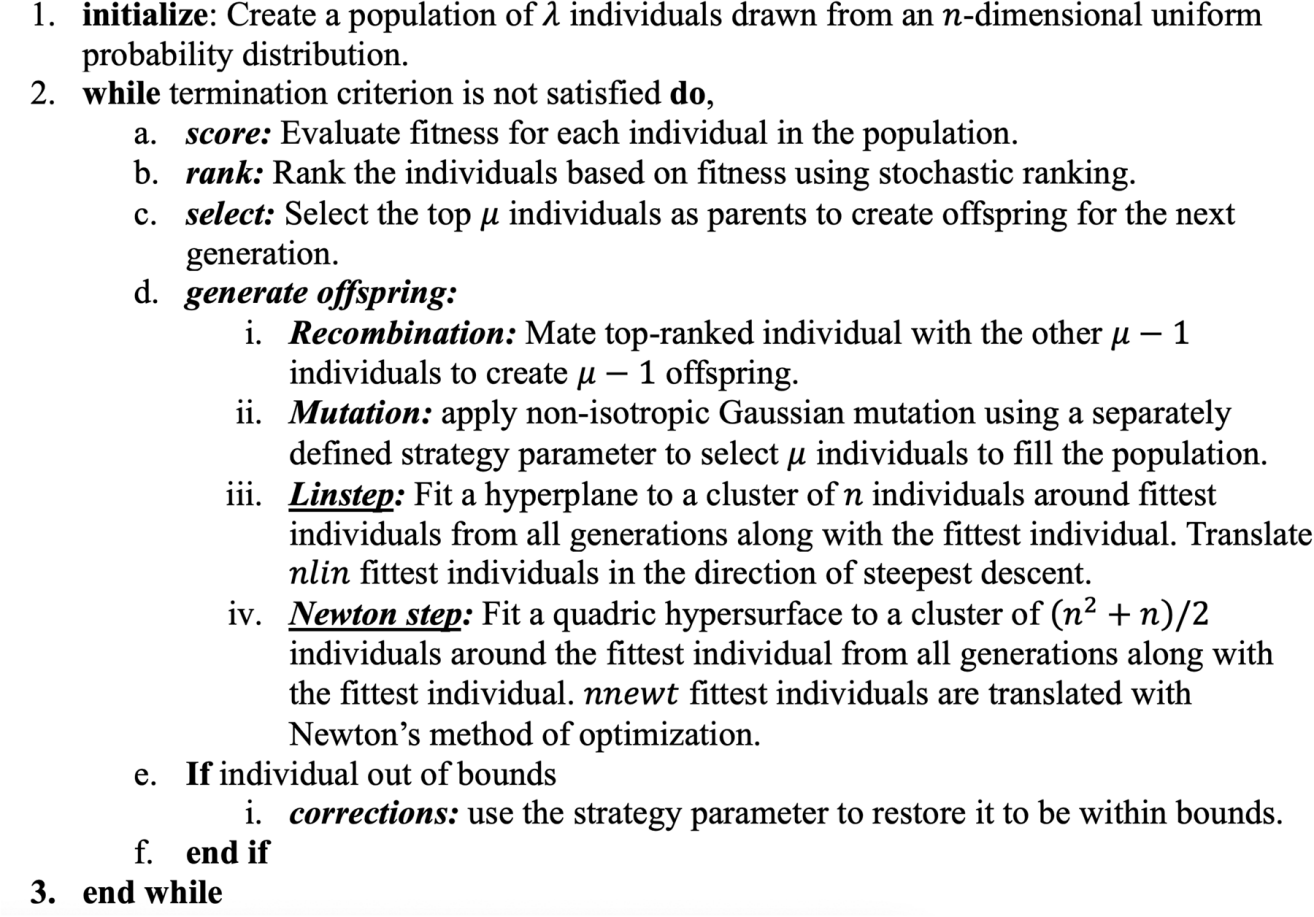
The ISRES+ algorithm. The ISRES+ algorithm initializes a random population of λ individuals. The algorithm then uses scoring and ranking strategies followed by selection of μ fittest individuals. To create a new generation, the algorithm uses recombination, mutation, Linstep, and Newton step to generate new offspring. Linstep and Newton step are strategies implemented only in ISRES+ (underscored) to improve the performance of the original ISRES. Finally, if the offspring is out of bounds, then the strategy parameter is used to restore it within bounds.

ISRES+ builds on the ISRES algorithm, proposed by Runnarson and Yao, for the minimization of continuous variable non-linear functions (Runarsson and Yao 2005). Figure 1 describes the ISRES+ algorithm, which builds on ISRES. The underscored strategies in Fig. 1 are only implemented in ISRES+, whereas all the other strategies are common to both ISRES and ISRES+. In the original ISRES algorithm, a population of λ individuals is initialized from an n-dimensional uniform distribution, where an individual is a set of n parameters that characterize the model. The population is then ranked using stochastic ranking by maintaining a balance between the objective and penalty functions in a way that it only slightly favors the feasible search space (which is defined as the region of the search space in which the penalties are satisfied). If it is an unconstrained optimization problem, then only the objective function is used for ranking. In every generation, $\mu \left(<\lambda \right)$ top-ranked individuals are selected for evolution to the next generation while the rest are discarded. The selected individuals produce offspring to populate the next generation by two methods.

In the first method, the top-ranked individual mates with the other parents to produce μ-1 offspring by the process of recombination according to the equation, where, $i=\left(2,\ldots ,\mu \right)$, g refers to the generation, and γ is the recombination parameter. In the second method, each of the selected parents are repeated rank-wise to fill the remaining λ-μ+1 population and the constituent parameters of an individual are subject to random mutations according to a gaussian probability distribution, according to the equation, where, $i=\left(\mu +1,\ldots ,\lambda \right)$, $j=\left(1,\ldots ,n\right)$, $N_{j}$ is a normally distributed random number for each parameter, and where $\eta_{i,j}$ is a strategy parameter which is essentially a measure of mean step sizes for each model parameter to take to maintain maximum progress to reach the function minimum, $\tau^{\prime }=\varphi /\sqrt{2n}$ is the learning rate for an individual, $\tau =\varphi /\sqrt{2\sqrt{n}}$ is the learning rate for each parameter, and $N\left(0,1\right)$ is a normally distributed random variable vector (Beyer 1995, Schwefel and Rudolph 1995). The variable φ is the expected rate of convergence and is usually set equal to 1. The mutation strategy is self-adaptative, as the strength of mutation is independent of the rest of the population and depends only on its parents’ strategy parameters, and is non-isotropic, as each parameter of each individual is multiplied by a distinct random number. At the end of each generation, mutation strength is reduced by exponential smoothing according to the equation, where $i=\left(1\ldots \lambda \right)$ and α is the smoothing factor (Runarsson 2002). The ratio of recombination contributions to that of mutation is kept at ∼1/7 to roughly ensure an equal probability of success in finding a fit individual by the two methods (Runarsson and Yao 2005).


(5) $$x_{i}^{g+1}=x_{i}^{g}+\gamma \left(x_{i}^{g}-x_{1}^{g}\right),$$

(6) $$x_{i}^{g+1}=\;x_{i}^{g}+\eta_{i,j}^{g+1}N_{j}\left(0,1\right),$$

(7) ηi,jg+1=ηi,jg exp⁡τ'N0,1 +τ Nj0,1,

(8) $$\eta_{i}^{g+1}=\eta_{i}^{g}+\alpha \left(\eta_{i}^{g+1}-\eta_{i}^{g}\right),$$

ISRES+ extends the original ISRES algorithm by obtaining directions to evolve the individuals in by fitting the fitness landscape in the local neighborhood of the best individual to either first- or second-order polynomials of the parameters. The process of evolution generates excess individuals that are discarded as the generations proceed. The idea is to use the additional information to better understand the fitness landscape and improve the search direction estimates. Linear least squares fit between the individuals and fitness values is performed using first- and second-order polynomials. The coefficients obtained are then correlated to a Taylor series expansion to obtain a pseudo gradient and a pseudo-Hessian. The gradient is used to perform a gradient-descent step and the Hessian is used to perform one iteration of the Newton’s method of function optimization.

To fit a first-order polynomial (hyperplane), a minimum of n+1 feasible individuals are required. First, all feasible individuals are sorted by Euclidean distance from the fittest one. The fittest individual along with the cluster of the closest n individuals around it are then used to fit a hyperplane, which is then used to determine the gradient v=df/dx for all individuals in the cluster, where f is the fitness and x is the parameter vector. The obtained gradient approximates the direction of the steepest descent. Individuals from the cluster are then translated along the steepest direction according to the equation, where $i=\left(\lambda -\mathrm{nlin}\right),..,\lambda$; nlin is the number of Linstep contributions to the population; $\beta_{\mathrm{lin}}\geq \;1$ and $d_{\mathrm{cluster}}$ are the diameter of the cluster bubble of individuals that were used to determine the gradient. The value of $\beta_{\mathrm{lin}}$ is generally close to 1. This method, henceforth called Linstep, is an approximate steepest descent step. (See Supplementary Section S2.1 for a detailed derivation of Linstep.)


(9) $$x_{i}^{g+1}=x_{i}^{g}-\beta_{\mathrm{lin}}d_{\mathrm{cluster}}v_{i},$$

To fit a second-order polynomial, the fittest individual along with a cluster of the closest $\left(n^{2}+n\right)/2$ individuals are used. The coefficients of the second-order polynomial are used to construct an approximate Hessian, ${H=d}^{2}f/dx^{2}$, and a new value of the gradient. These approximations are then used to perform a single iteration of Newton’s method of function optimization on the fittest individual, according to the equation, where $i=\left(\lambda -\mathrm{nlin}-\mathrm{nnewt}\right),.,\left(\lambda -\mathrm{nnewt}\right)$ and $0\;<\;\beta_{\mathrm{newt}}\leq \;1$; nnewt is the number of Newton step contributions to the population. This method will henceforth be called *Newton step*. In each case, a minimum number of individuals are chosen to determine these directions and if the resulting matrix of the gradient or the Hessian is ill-conditioned, then more individuals are used. (See Supplementary Section S2.1 for a detailed derivation of Newton step.)


(10) $$x_{i}^{g+1}=x_{i}^{g}-\beta_{\mathrm{newt}}H^{-1}v_{i},$$

Note that, while nlin of the top-ranked individuals are translated in the direction of the approximate steepest descent in Linstep with a fixed value of $\beta_{\mathrm{lin}}$, only the fittest individual is used to generate Newton step contributions since all individuals for which the Hessian and the gradient are computed moved to the same point (see Supplementary Section S2). Multiple contributions (nnewt) are made by using random values for $\beta_{\mathrm{newt}}$. Since Linstep fits a hyperplane, its strategy to generate new individuals is spread across multiple fit individuals used to yield the fit. Since Newton step fits a quadric hypersurface around the best-fit individual, its strategy to generate new individuals is to perform a Newton’s method of function optimization step using the best-fit individual. In this case, multiple contributions are made varying the step size. The individuals contributed by Linstep and Newton step are added to the population at the expense of mutation contributions around the least fit parents according to the ES, thus maintaining the size of the population λ. We chose a small value of nlin to make sure the algorithm is not biased towards the fittest individual in the earlier generations and nnewt equal to 1 as a full Newton step leads all individuals considered in the Hessian calculation to move to the same point. The population continues to evolve by each of the four methods for a user-defined number of generations. The algorithm outputs the fittest individual found in all generations and the value of its objective function, along with some statistics about the evolution.

## 4 Results

We tested the performance of ISRES+ in estimating parameters for three ODE-based systems biology models and compared it with ISRES. For the ES part of the algorithm, we used the recommendations for the settings of the hyperparameters from the original ISRES algorithm proposed by Runnarson and Yao in all three models (Runarsson and Yao 2005). For every combination of hyperparameters, we ran each of the algorithms at least 50 times. The performance of ISRES+ (blue) over successive generations and the inverse fitness (error) of the fittest individual over all generations was compared with the original ISRES (red) algorithm for all three models.

**Figure 2.**
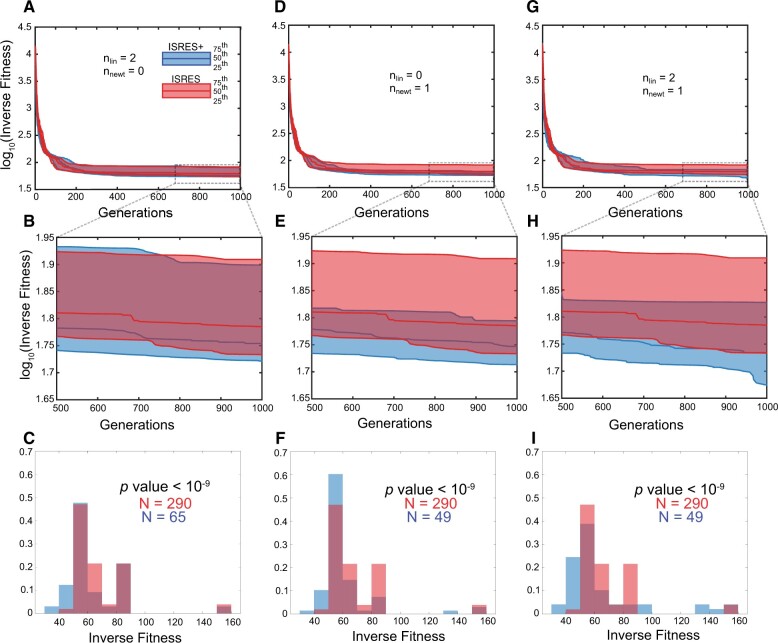
ISRES+ v/s ISRES comparison for the Dl/Cact model. (A) The three plot lines represent 75th, 50th, and 25th percentile of all the independent simulations (*N* > 50). The plot indicates the $\log_{10}\mathrm{inverse}\;\mathrm{fitness}$ across generations. (B) The plot in (A) was zoomed-in to compare the plot lines in the last 500 generations. (A and B) Only Linstep (nlin = 2) is active throughout the duration of the simulation. ISRES+ performs better than ISRES as the 25th and 50th percentile is lower for ISRES+ than ISRES. (C) Histogram plot of the inverse fitness from all independent simulations. ISRES+ has higher number of data points in the lower inverse fitness region compared with ISRES. (D and E) Only Newton step is active throughout the duration of the simulation. The 25th, 50th, and 75th percentiles indicate that ISRES+ performs better than ISRES. The distribution of $\log_{10}\mathrm{inverse}\;\mathrm{fitness}$ is tighter for ISRES+ which means on average ISRES+ performs better than ISRES. (F) The histogram indicates that a higher number of solutions have a lower inverse fitness when ISRES+ was used instead of ISRES. (G and H) Both Linstep and Newton step remain active throughout the duration of the simulation. ISRES+ performs better than ISRES as the distribution is narrower compared with ISRES and all three plot lines are lower than that of ISRES. (I) More ISRES+ runs have a lower inverse fitness than ISRES (*p-*value was calculated using Wilcoxon test in MATLAB).

For the Dorsal/Cactus (Dl/Cact) model (see Supplementary Material for additional details on the Dl/Cact model), the number of Linstep and Newton step contributions to the population were varied and the performance of ISRES+ was compared with ISRES. In Fig. 2, the inverse fitness (minimum value of error obtained by evaluating the objective function) for the fittest individual across at least 50 independent simulations is plotted in the first two rows of plots while the last row of plots are histograms of the inverse fitness best individual obtained at the end of the simulation. The three plotlines in the first two rows of plots indicate the 25th, 50th, and 75th percentile of the inverse fitness for every generation across the >50 independent simulations. The histogram plots, however, represent the inverse fitness of the fittest individual over all generations normalized by probability for all independent simulations of ISRES and ISRES+. In Fig. 2A–C, only Linstep is active during the entire duration of the simulation and contributes two individuals to the population in every generation, while in Fig. 2D–F, only Newton step is active and contributes one individual. In Fig. 2G–I, both Linstep and Newton step are active, with the former contributes two individuals and the latter contributes one individual to the population in every generation. Figure 2A and B indicates that when only Linstep is on, the ISRES+ algorithm performs slightly better than ISRES at the 25th and 50th percentile. The histogram of the inverse fitness in Fig. 2C indicates that, across more than 50 independent runs, ISRES+ performs significantly better (*p-*value calculated using Wilcoxon sum test in MATLAB is less than 10^−9^) than ISRES when only Linstep is on. As seen in Fig. 2D–F, when only Newton step is on, the final performance improves only slightly, as seen by comparing the median line between Fig. 2E and B. However, there is a noticeable change in the final distribution of individuals that forms a much tighter cluster as also demonstrated by the histograms. When both Linstep ($n_{\mathrm{lin}}=2$) and Newton step ($n_{\mathrm{newt}}=1$) contribute to the population, the breadth of the final distribution is intermediate to the cases when only one of the two methods is active but is better than either case. While the Newton step-only case certainly has a tighter distribution than this case, the trend from G=500 indicates that the lower percentile curves are getting better. This distribution of inverse fitness of the fittest individual over all generations is significantly better (*p-*value calculated using Wilcoxon sum test in MATLAB is less than 10^−9^) for ISRES+ than ISRES as can also be seen in the histograms, that are plotted over all the results (Fig. 2F and I). Note that the 75th percentile plotline for ISRES+ follows that of ISRES, when only Linstep is active (Fig. 2A–C), and the plotline converges markedly at *G* ≅ 300 when only Newton step is active. When both Linstep and Newton step are active, the converging behavior is observed later at *G* ≅ 500. For the Dl/Cact model, while Newton step seems to assist in the converging behavior that leads to a tighter final distribution. (Fig. 2F), both methods are required to also obtain a better final distribution (Fig. 2I). Thus, for the Dl/Cact model, Newton step-only strategy performs best, while Linstep-only and both Linstep and Newton step still outperform ISRES even though they have a wider distribution of inverse fitness. Note that the Dl/Cact model is defined as a constrained optimization problem and the constraint handling strategy of stochastic ranking is retained within ISRES+ as well. The plots shown are only over individuals that satisfy all the constraints. In summary, for the Dl/Cact model, Newton step-only mode results in a tighter distribution (Fig. 2B, E, and H) whereas both Linstep and Newton step active mode results in a higher number of fittest individuals with a lower inverse fitness (Fig. 2C, F, and I).

**Figure 3.**
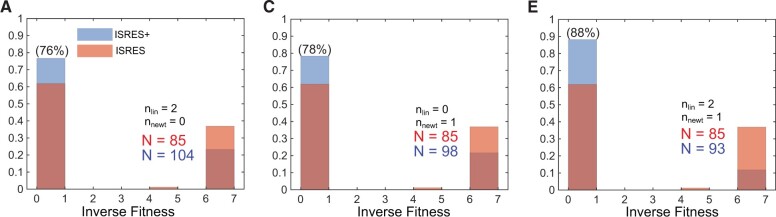
ISRES+ v/s ISRES comparison for the Smad signaling model. (A) ISRES+ was run with only Linstep active throughout. The histogram plot of the inverse fitness from all independent runs shows that ISRES+ found the lower minima in ∼76% runs compared with ISRES which found it in ∼60% runs. (B) ISRES+ was run with only Newton step active throughout. The histogram plot of the inverse fitness from all independent runs shows that ISRES+ found the lower minima in ∼78% runs compared with ISRES which found it in ∼60% runs. (C) ISRES+ was run with both Linstep and Newton step active throughout. The histogram plot of the inverse fitness from all independent runs shows that ISRES+ found the lower minima in ∼88% runs compared with ISRES which found it in ∼60% runs.

For the Smad signaling model (see Supplementary Material for additional details on the Smad model), we found that the model had at least two local minima, one around inverse fitness ≈ 0.5 and the other around inverse fitness ≈ 6. From Fig. 3A, C, and E, we find that both algorithms mostly converge to either one of the two minima fairly quickly (by G<100). In Fig. 3B, D, and F, the histograms of the final distribution of error values normalized by probability are plotted. In the Linstep-only mode, ISRES+ finds a better solution more often than ISRES, such that 76% of all ISRES+ runs converge to the lower minima as opposed to ∼60% for ISRES. In the Newton step-only mode, ISRES+ performs slightly better than the Linstep-only mode and finds the lower minima in 78% of the runs. When both Linstep and Newton step are active, ISRES+ outperforms ISRES by converging to the lower minima in 88% of the runs. For the Smad signaling model, both strategies, Linstep and Newton step, perform well individually, but together they perform even better.

**Figure 4.**
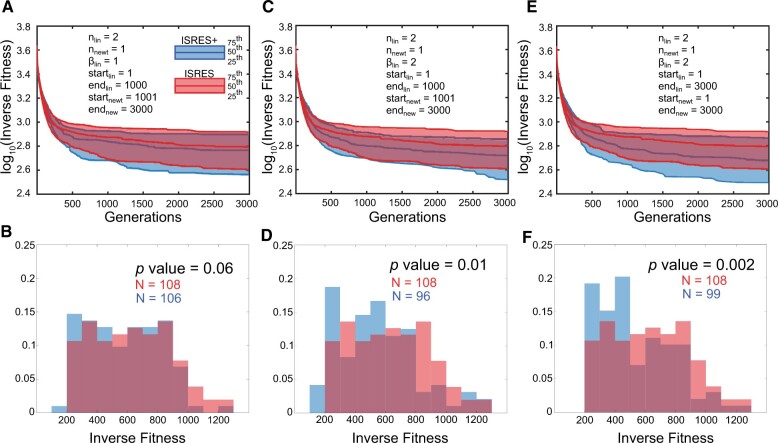
ISRES+ v/s ISRES comparisons for the gap gene circuit model. (A) The three plot lines represent 75th, 50th, and 25th percentile of all the independent simulations (*N* > 50). The plot indicates the inverse fitness across generations. (B) Histogram plot of the inverse fitness of the fittest individual over all generations from all independent simulations. (A and B) ISRES+ was run with Linstep active for the first 1000 generations and Newton step active from 1000th to 3000th generations. There is a marginal improvement in the 25th and the 50th. The histogram indicates that ISRES+ found solutions that fit the model slightly better than ISRES. (C and D) ISRES+ has the same hyperparameters as (A and B) except $\beta_{\mathrm{lin}}$, which is 2 in this case. This allows the ISRES+ to perform significantly better than ISRES at all three plot lines and the histogram also indicates that ISRES+ found better fits to the model compared with ISRES. (E and F) ISRES+ was run with Linstep and Newton step active throughout with a $\beta_{\mathrm{lin}}=2$. ISRES+ has a lower 25th, 50th, and 75th percentile than ISRES although the distribution is wider. The histogram shows that ISRES+ found more solutions that were better than ISRES more often (*p-*value was calculated using a modified *t*-test).

The three strategies discussed above: Linstep-only, Newton step-only, and both Linstep and Newton step active throughout perform well for the Dl/Cact and Smad signaling model, but for the gap gene circuit model (see Supplementary Section S2 for additional details on the gap gene circuit model), they yield comparable results for ISRES and ISRES+ (see Supplementary Section S4). So, we varied the generation at which Linstep and Newton step are switched on or off (Fig. 3). Note that in all configurations of hyperparameters, $n_{\mathrm{lin}}=2\;$and $n_{\mathrm{newt}}=1$. In Fig. 4A and B, Linstep is active from G=1-1000, while Newton step is active from G=1001-3000. Since the model is relatively more complex, the idea was to provide the evolutionary part of the algorithm enough power in the search. Linstep contributions are expected to dominate earlier in the search strategy when the population is more randomly distributed, while Newton step is expected to perform better later since the population may be expected to be closer to a minimum. For the configuration in Fig. 4A and B, we find only a small improvement over ISRES. Since Linstep does not have a defined step size like Newton step (see Algorithm section), we varied the step size (by varying $\beta_{\mathrm{lin}}$) to tune the algorithm even further. Note that *β*_lin_ is the Linstep parameter that controls the magnitude of the gradient-descent step with respect to the diameter of the cluster bubble formed by the individuals used to determine the gradient. A value of this parameter > 1 indicates that the magnitude of the gradient descent step is larger than the diameter of the cluster bubble. While theoretically, any value of *β*_lin_ ≥ 1 would work, practically, a value not too much higher than one would be most appropriate to protect the solution from deviating too far away from the bubble into uncharted terrain. When *β*_lin_ = 2, the same configuration performs much better as shown in Fig. 4C and D. In Fig. 4E and F, when *β*_lin_ = 2 and when both Linstep and Newton step are active during the entire duration of evolution, ISRES+ performs significantly better than ISRES. However, for other values of *β*_lin_ that we tested, the performance of ISRES+ was in most cases comparable to ISRES (see Supplementary Section S5).

In summary, Newton step performs better than Linstep for the Dl/Cact model. Both Linstep and Newton step have a comparable performance for the Smad signaling model, but having both Linstep and Newton step active throughout yields a better performance. The three strategies that work well for Dl/Cact and Smad signaling model underperform for the gap gene model while switching Linstep off early and switching Newton step on late and having a *β*_lin_ = 2 performs the best.

In general, Linstep and Newton step help ISRES+ perform better than ISRES. The configuration of hyperparameters for the three models discussed here are just recommendations based on extensive tests performed on a trial-and-error basis. The complexity of the models in systems biology precludes us from generalizing a set of hyperparameters that would help ISRES+ always perform better than ISRES.

Overall, we find that adding a few contributions from linear least square fit-based methods into the population leads to obtaining a more fit individual across all the biological models tested. Also, we find that the probability of obtaining a more fit individual over several independent runs is higher. This is generally true for a wide range of hyperparameter values and requires both Linstep and Newton step to obtain the best results.

## 5 Discussion and conclusions

Evolutionary algorithms like ISRES are widely applied in systems biology to estimate model parameters (Ashyraliyev *et al.* 2009b, Jostins and Jaeger 2010, Crombach *et al.* 2012, Kozlov *et al.* 2012, Spirov and Holloway 2013, Lobo and Levin 2015, O’Connell and Reeves 2015, Liu *et al.* 2016, Filkova *et al.* 2019, Maeda *et al.* 2019). ISRES uses randomly generated parameter sets, or “individuals,” to evaluate the objective function and binary constraints to select fitter individuals and generate offspring through recombination and mutation, finally evolving through recombination and mutation into the fittest individual. ISRES creates a large population of individuals, thus generating a large amount of information, but only a small amount of it is utilized. In recombination, the fittest individual shares information in a pair-wise fashion with the next top *μ*−1 individuals, while in mutation, no information is shared at all. Thus, in these conventional evolutionary strategies, information is partially shared within a generation to create fitter individuals, but they fail to utilize the abundance of information that is generated about the fitness landscape across generations.

Our modified algorithm, ISRES+, seeks to take advantage of this information by understanding the features of the fitness landscape by sharing information, potentially, between all individuals explored up until that point in evolution. To do this, ISRES+ employs two gradient-based strategies: *Linstep* and *Newton step*, to understand the features of the fitness landscape by sharing information between individuals in all the previous generations to generate new offspring. These individuals are chosen based on their proximity to the fittest individual and not generation or lineage, allowing ISRES+ to share information between a lot more individuals, which helps probe the fitness landscape around the fittest individual.

The first strategy, Linstep, is a first-order linear least squares fit method which generates offspring by approximating the structure of the fitness landscape by fitting a hyperplane through O(n) individuals. In Linstep, a gradient descent step is performed on a small number of individuals (1 − 3) using the direction obtained from a cluster of individuals in the immediate vicinity of the fittest individual, which is analogous to a first-order Taylor series expansion. The magnitude of the step is chosen as a fraction of the diameter of the cluster. Linstep is expected to work better in the earlier generations when the best parameter set may be far from a minimum, in which case only a general direction of descent is needed. On the other hand, in later generations, when the population may be closer to a minimum, Linstep could potentially overshoot a minimum basin in a phenomenon known as gradient hemistitching. In fact, Linstep is likely more prone to this issue than traditional gradient descent methods, as the parameter sets that are used to generate the hyperplane approximation may lie on either side of a minimum basin, which would result in a highly inaccurate gradient approximation.

The second strategy, the Newton step is a second-order linear least squares fit method which generates new offspring by approximating the structure of the fitness landscape around the $O(n^{2})$ individuals around the fittest individual in every generation by a quadric hypersurface. In Newton step, a single iteration of Newton’s method of function optimization is performed by fitting a cluster of individuals to a quadric hypersurface paraboloid in n dimensions. The polynomial coefficients of the paraboloid are then used to construct an approximate Hessian and a new value of the gradient for each individual, which is analogous to a second-order Taylor series expansion. Newton’s step is executed on a handful of individuals (1–3) that were used in the fitting procedure. It is important to note that performing a full Newton’s step ($\beta_{\mathrm{newt}}=1$ in Equation 10) takes all individuals considered in the Hessian calculation to the same point in search space. Therefore, this method could either contribute a single individual from a full Newton step or several with varying step sizes. In all of our simulations, we ensured that Newton’s method contributed one individual to the population every generation, Newton’s step is expected to work better in later generations when the algorithm is close to convergence and Newton step could more precisely direct the search to a minimum.

These intelligent search strategies enable ISRES+ to probe the fitness landscape using gradient-based optimization techniques while retaining the advantages of evolutionary strategies.

Since the fitness landscape of complex models in systems biology is expected to be convoluted, we do not expect Linstep and Newton step to make significant contributions in every generation. During the search process, if the evolutionary methods appear to get “stuck,” it will lead to the creation of several individuals within a bubble of small diameter in the n-dimensional parameter space. In such a case, Linstep and Newton step are silenced until a new best-fit individual is found by the stochastic evolutionary methods. Since the landscape is not known beforehand, we perform Linstep and Newton step in every generation, rather than deciding arbitrarily on what criterion of distance qualifies as approximate enough to activate these methods. It is important to note that there is no additional overhead of calculating the gradient or the Hessian, which would require O(n) and $O(n^{2})$ functional computations, respectively, to construct the approximation. Instead, the fitness landscape that has already been explored is used to get approximate values of the gradient and the Hessian. Thus, the only additional overhead involved is in performing the matrix back-substitution to calculate the parameters characterizing the linear and quadratic equations. The benefit becomes more apparent in complex systems, like that of a systems biology model, wherein the cost of computing the function is significantly higher than the cost of the matrix back-substitution.

In all three systems biology models, we find that small contributions from Linstep and Newton step to the population bias the search in a way that it converges to a more fit region of parameter space faster and more often. Also, the final distribution of the fittest individuals obtained across multiple independent runs is much tighter compared with ISRES. While one might generally expect that once enough individuals are created (within a handful of generations with our population size, λ=150), Newton step might outperform Linstep since it is a second-order method. However, we found that, across all three models, having both methods turned on had the highest probability of obtaining the best solutions consistently over several independent runs. This may be attributed to the fact that the fitness landscape of the multiparameter systems biology models is complex and the region around the fittest individual may be best approximated by a hyperplane or by a quadric hypersurface. Most of the initial gains may be attributed to Linstep, while Newton step seems to be responsible for ensuring that the algorithm converges to the same region in function space over multiple runs. Models in systems biology are complex, making it difficult to generalize a combination of hyperparameters that help improve the performance of ISRES+, but generally, small contributions of the two methods to the population in every generation lead to better overall performance. With our suggested settings of hyperparameters: *n*_lin_ = 2, *n*_newt_ = 1, and *β*_lin_ = 2, ISRES+ generally outperforms ISRES. Even when this is not the case ISRES+ rarely performs worse, and given the wall clock times are indistinguishable, ISRES+ only improves upon ISRES. This may be attributed to the fact that Linstep and Newton step attempt to direct the strategy to search around the fittest individual found so far, even if the ES methods have shifted focus to exploring other regions that might neither have lower penalty values nor lower objective function values. Since the methods focus on the landscape local to the fittest individual in all generations, there is mixing of information not only from within the population but also between generations. The relative success of these methods may be attributed to the fact that the entire evolutionary history is used to develop a better understanding of the functional landscape around the fittest individual found so far and optimal search directions are obtained in a deterministic manner.

## Supplementary Material

btad403_Supplementary_Data

## Data Availability

The data underlying this article are available at Github at https://github.com/gtreeves/isres-plus-bandodkar-2022 and in the Zenodo repository at https://doi.org/10.5281/zenodo.8092353.
