# Supplementary material for: ISRES+: an improved evolutionary strategy for function minimization to estimate the free parameters of systems biology models
Source: Bioinformatics. 2023 Jun 24;39(7):btad403. doi: 10.1093/bioinformatics/btad403 (PMC10323169; doi:10.1093/bioinformatics/btad403)
Supplement: btad403_Supplementary_Data [file btad403_supplementary_data.docx]

# Model Description

## Dl/Cact model

### Description

The Dl/Cact model describes the formation of the Dorsal (Dl) gradient that patterns the dorsal-ventral (DV) axis in early *Drosophila* embryos (Kanodia et al. 2009; O’Connell and Reeves 2015; Carrell et al. 2017; Al Asafen et al. 2020). Dorsal is a widely studied maternal morphogen that is initially uniformly expressed in the early embryo. Dl binds to another maternally deposited protein, Cactus (Cact), in the cytoplasm that inactivates it. The Dl/Cact complex binds to Toll receptors resulting in its dissociation leading to free Dl. Free Dl can enter the nucleus and regulate gene expression. Since Toll is distributed asymmetrically along the DV axis, the Dl gradient is formed with high concentrations along the dorsal midline. The Dl gradient then activates genes in a concentration-dependent manner and patterns the DV axis into three tissue types – mesoderm, neuroectoderm, and dorsal ectoderm.

### Mathematical model

The mathematical equations of the model may be represented as follows,

| $\frac{d\left[ V_{nuc}C_{d,nuc}^{h} \right]}{dt}= A_{nuc}\left( k_{in,d}C_{d,cyt}^{h}-k_{out,d}C_{d,nuc}^{h} \right) -V_{nuc}\left( k_{b}C_{d,nuc}^{h}C_{c,nuc}^{h} \right)$ | (1) |
| --- | --- |
| $\frac{d\left[ V_{cyt}C_{d,cyt}^{h} \right]}{dt}= A_{cyt}\Gamma_{d}\left( C_{d,cyt}^{h-1}-2C_{d,cyt}^{h}+ C_{d,cyt}^{h+1} \right) +V_{cyt}\left( \frac{k_{d}\left( x \right)C_{dc,cyt}^{h}}{\kappa+C_{dc,cyt}^{h}}- k_{b}C_{d,cyt}^{h}C_{c,cyt}^{h} \right) -A_{nuc}\left( k_{in,d}C_{d,cyt}^{h}-k_{out,d}C_{d,nuc}^{h} \right)$ | (2) |
| $\frac{d\left[ V_{nuc}C_{dc,nuc}^{h} \right]}{dt}= A_{nuc}\left( k_{in,dc}C_{dc,cyt}^{h}-k_{out,dc}C_{dc,nuc}^{h} \right) + V_{nuc}\left( k_{b}C_{d,nuc}^{h}C_{c,nuc}^{h} \right)$ | (3) |
| $\frac{d\left[ V_{cyt}C_{dc,cyt}^{h} \right]}{dt}= A_{cyt}\Gamma_{dc}\left( C_{dc,cyt}^{h-1}-2C_{dc,cyt}^{h}+ C_{dc,cyt}^{h+1} \right) -V_{cyt}\left( \frac{k_{d}\left( x \right)C_{dc,cyt}^{h}}{\kappa+C_{dc,cyt}^{h}}- k_{b}C_{d,cyt}^{h}C_{c,cyt}^{h} \right) -A_{nuc}\left( k_{in,dc}C_{dc,cyt}^{h}-k_{out,dc}C_{dc,nuc}^{h} \right)$ | (4) |
| $\frac{d\left[ V_{nuc}C_{c,nuc}^{h} \right]}{dt}= A_{nuc}\left( k_{in,c}C_{c,cyt}^{h}-k_{out,c}C_{c,nuc}^{h} \right) -V_{nuc}\left( k_{b}C_{d,nuc}^{h}C_{c,nuc}^{h} \right)$ | (5) |
| $\frac{d\left[ V_{cyt}C_{c,cyt}^{h} \right]}{dt}= A_{cyt}\Gamma_{c}\left( C_{c,cyt}^{h-1}-2C_{c,cyt}^{h}+ C_{c,cyt}^{h+1} \right) +V_{cyt}\left( \frac{k_{d}\left( x \right)C_{dc,cyt}^{h}}{\kappa+C_{dc,cyt}^{h}}- k_{b}C_{d,cyt}^{h}C_{c,cyt}^{h}- k_{deg}C_{c,cyt}^{h} \right) -A_{nuc}\left( k_{in,c}C_{c,cyt}^{h}-k_{out,c}C_{c,nuc}^{h} \right)+P_{c}$ | (6) |

where, subscripts *nuc* and *cyt* represent nucleus and cytoplasm; subscripts *d*, *c*, and *dc* represent the species Dl, Cact and the Dl/Cact complex; A, V, and C represent area, volume, and concentrations; $\Gamma$ represents intercompartmental exchange rates; $k_{d}\left( x \right)=k_{d}^{max}\exp\left( -\frac{1}{2}\left( \frac{x}{\phi} \right)^{2} \right)$, $k_{b}$, $k_{deg}$, and $\kappa$ represent gaussian Toll-mediated rate constant, the Dl/Cact binding constant, the degradation rate constant for Cact, and Michaelis Menten constant for the dissociation of Dl/Cact complex respectively; $k_{in}$ and $k_{out}$ represent nuclear import and export rates respectively; and $P_{c}$ represents rate of production of Cact.

### Simulation Conditions

The Dl/Cact model was run according to O’Connell and Reeves, 2015, where a similar model was first introduced. Live imaging of Dl was used as the experimental data and the corresponding spatio-temporal grid generated was implemented in the model. Note that the number of spatio-temporal points change with nuclear cycles. The model data was rescaled to that of the experimental data to ensure appropriate error calculations.

### Model Results

The following Fig S1 shows the concentration of Dl ($C_{d,nuc}+C_{d,cyt}$) plotted against simulated data at the ventral ($x=0$) and dorsal ($x=1$) midlines for one of the highly fit parameter sets.

| 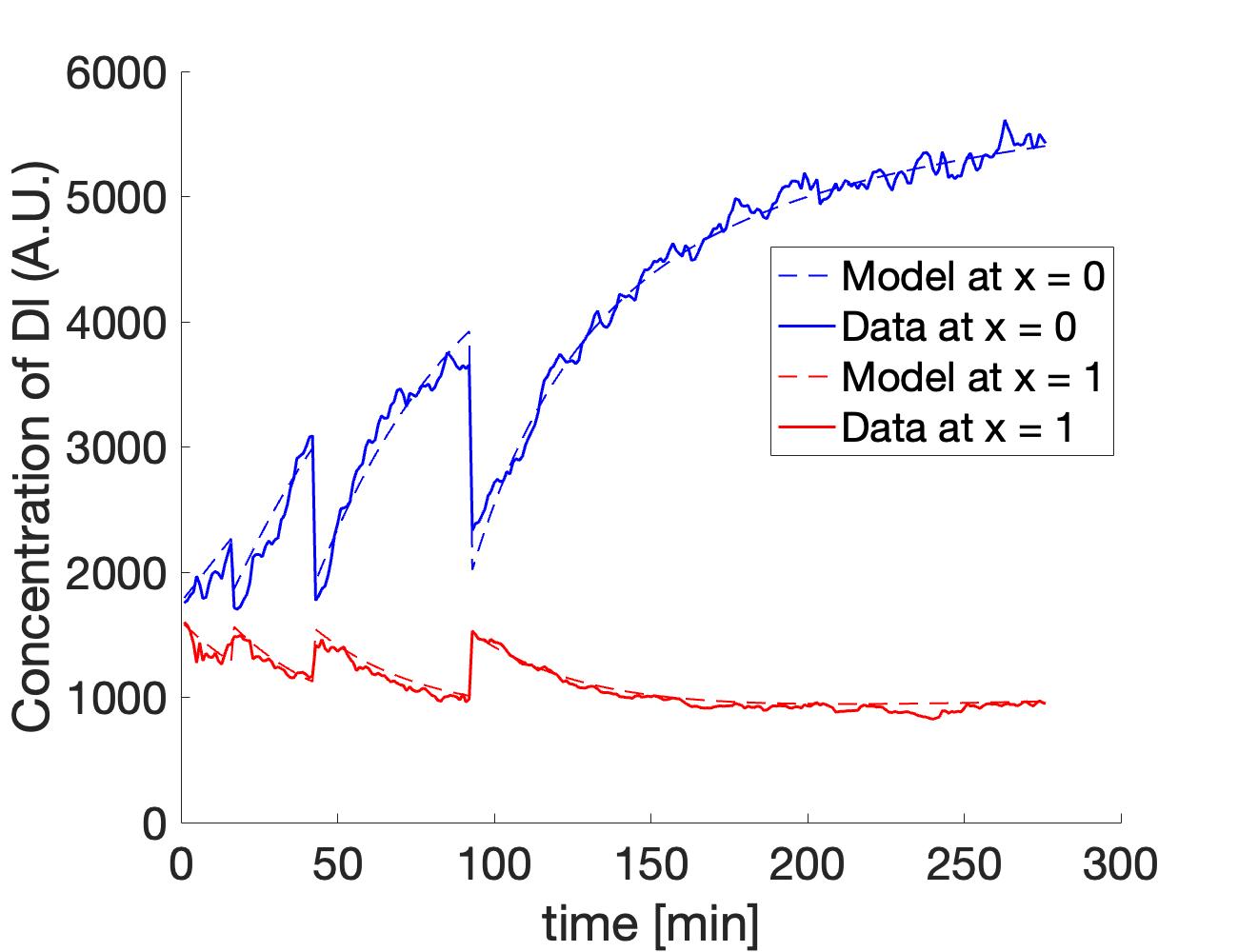  **Fig S1. Model and experimental data plotted for the Dl/Cact model.** The plot represents Dl concentration profile from nuclear cycle 11 to 14 at the dorsal and ventral midlines of the early Drosophila embryo. |
| --- |

## Smad signaling model.

### Description

Transforming growth factor beta (TGF-β), the founding member of the TGF-β super-family of transforming growth factors, induces Smad signaling, which controls developmental processes such as embryogenesis, proliferation, and apoptosis throughout the body (Schuster and Krieglstein 2002; Huang and Huang 2005; Kitisin et al. 2007; Aashaq et al. 2022). TGF-β triggers a signaling cascade by binding to and activating the receptor. Activated receptors phosphorylate Smad2, the receptor-regulated Smad (R-Smad). The phosphorylated-Smad2 (pSmad2) forms a heteromeric complex with Smad4, the common Smad (Co-Smad). These complexes translocate to the nucleus and regulate gene expression. All the Smad species move dynamically between the cytoplasm and the nucleus, so in the presence of TGF-β signaling, Smad2, phosphor-Smad2, Smad2-Smad2, and Smad2-Smad4 are present in the nucleus (Schmierer and Hill 2007; Massagué 2012). In the absence of TGF-β signaling, Smad2 is inactive and does not form any heteromeric complexes, so the only Smad species in the nucleus are Smad2 and Smad4.

Schmierer et al. 2008, visualized Smad nucleo-cytoplasmic dynamics with EGFP-tagged-Smad2 through live imaging and quantified the accumulation of Smad species in the nucleus. They activated the Smad signaling cascade by inducing receptor activation which led to the accumulation of Smad species in the nucleus. Then after 45 mins, they added an inhibitor that deactivated the pathway, resulting in a decumulation of Smad species in the nucleus. They fit this fluorescence data to an ODE-based mathematical model and found that the Smad2-Smad2 and Smad2-Smad4 complexes have a higher import rate (complex import factor (CIF) > 1) to enable nuclear accumulation of Smad2 (Schmierer et al., 2008).

### Mathematical model

We used the Retention/Enhanced Complex Import (RECI) model reported by Schmierer et al. (2008)., which has 25 state variables and 10 variable parameters (Schmierer et al. 2008). Schmierer et al. estimated the values of four parameters and optimized the other six. In our simulations, we optimized all 10 parameters.

| $\frac{d\left[ R \right]}{\mathrm{dt}}=-k_{TGF-}\left[ R \right]\left[ TGF- \right]$ | () |
| --- | --- |
| $\frac{d\left[ \mathrm{TG}F-\beta\right]}{\mathrm{dt}}=-k_{TGF-}\left[ R \right]\left[ TGF- \right]$ | () |
| $\frac{d\left[ R^{\mathrm{act}} \right]}{\mathrm{dt}}=k_{TGF-}\left[ R \right]\left[ TGF- \right]-k_{\mathrm{onSB}}\left[ R^{\mathrm{act}} \right]\left[ \mathrm{SB} \right]+k_{\mathrm{offSB}}\left[ R^{\mathrm{inact}} \right]$ | () |
| $\frac{d\left[ R^{\mathrm{inact}} \right]}{\mathrm{dt}}=k_{\mathrm{onSB}}\left[ R^{\mathrm{act}} \right]\left[ \mathrm{SB} \right]-k_{\mathrm{offSB}}\left[ R^{\mathrm{inact}} \right]$ | () |
| $\frac{d\left[ \mathrm{SB} \right]}{\mathrm{dt}}=k_{\mathrm{offSB}}\left[ R^{\mathrm{inact}} \right]-k_{\mathrm{onSB}}\left[ R^{\mathrm{act}} \right]\left[ \mathrm{SB} \right]$ | () |
| $\frac{d\left[ S2 \right]_{c}}{\mathrm{dt}}=k_{\mathrm{ex}}\left[ S2 \right]_{n}-k_{\mathrm{in}}\left[ S2 \right]_{c}-k_{\mathrm{phos}}\left[ S2 \right]_{c}\left[ R^{\mathrm{act}} \right]$ | () |
| $\frac{d\left[ G \right]_{c}}{\mathrm{dt}}=k_{\mathrm{ex}}\left[ G \right]_{n}-k_{\mathrm{in}}\left[ G \right]_{c}-k_{\mathrm{phos}}\left[ G \right]_{c}\left[ R^{\mathrm{act}} \right]$ | () |
| $\frac{d\left[ pS2 \right]_{c}}{\mathrm{dt}}=k_{\mathrm{ex}}\left[ pS2 \right]_{n}-k_{\mathrm{in}}\left[ pS2 \right]_{c}+k_{\mathrm{phos}}\left[ S2 \right]_{c}\left[ R^{\mathrm{act}} \right]-k_{\mathrm{on}}\left[ pS2 \right]_{c}\left( \left[ S4 \right]_{c}+2\left[ pS2 \right]_{c}+\left[ \mathrm{pG} \right]_{c} \right)+k_{\mathrm{off}}\left( \left[ S24 \right]_{c}+2\left[ S22 \right]_{c}+\left[ G2 \right]_{c} \right)$ | () |
| $\frac{d\left[ \mathrm{pG} \right]_{c}}{\mathrm{dt}}=k_{\mathrm{ex}}\left[ \mathrm{pG} \right]_{n}-k_{\mathrm{in}}\left[ \mathrm{pG} \right]_{c}+k_{\mathrm{phos}}\left[ G \right]_{c}\left[ R^{\mathrm{act}} \right]-k_{\mathrm{on}}\left[ \mathrm{pG} \right]_{c}\left( \left[ S4 \right]_{c}+2\left[ pS2 \right]_{c}+\left[ \mathrm{pG} \right]_{c} \right)+k_{\mathrm{off}}\left( \left[ G4 \right]_{c}+2\left[ G2 \right]_{c}+\left[ \mathrm{GG} \right]_{c} \right)$ | () |
| $\frac{d\left[ S4 \right]_{c}}{\mathrm{dt}}=k_{\mathrm{in}}\left[ S4 \right]_{n}-k_{\mathrm{in}}\left[ S4 \right]_{c}-k_{\mathrm{on}}\left[ S4 \right]_{c}\left( \left[ pS2 \right]_{c}+\left[ \mathrm{pG} \right]_{c} \right)+k_{\mathrm{off}}\left( \left[ S24 \right]_{c}+\left[ G4 \right]_{c} \right)$ | () |
| $\frac{d\left[ S24 \right]_{c}}{\mathrm{dt}}=k_{\mathrm{on}}\left[ pS2 \right]_{c}\left[ S4 \right]_{c}-k_{\mathrm{off}}\left[ S24 \right]_{c}-k_{\mathrm{in}}\mathrm{CIF}\left[ S24 \right]_{c}$ | () |
| $\frac{d\left[ G4 \right]_{c}}{\mathrm{dt}}=k_{\mathrm{on}}\left[ \mathrm{pG} \right]_{c}\left[ S4 \right]_{c}-k_{\mathrm{off}}\left[ G4 \right]_{c}-k_{\mathrm{in}}\mathrm{CIF}\left[ G4 \right]_{c}$ | () |
| $\frac{d\left[ S22 \right]_{c}}{\mathrm{dt}}=k_{\mathrm{on}}\left[ pS2 \right]_{c}^{2}-k_{\mathrm{off}}\left[ S22 \right]_{c}-k_{\mathrm{in}}\mathrm{CIF}\left[ S22 \right]_{c}$ | () |
| $\frac{d\left[ G2 \right]_{c}}{\mathrm{dt}}=k_{\mathrm{on}}\left[ \mathrm{pG} \right]_{c}\left[ pS2 \right]_{c}-k_{\mathrm{off}}\left[ G2 \right]_{c}-k_{\mathrm{in}}\mathrm{CIF}\left[ G2 \right]_{c}$ | () |
| $\frac{d\left[ \mathrm{GG} \right]_{c}}{\mathrm{dt}}=k_{\mathrm{on}}\left[ \mathrm{pG} \right]_{c}^{2}-k_{\mathrm{off}}\left[ \mathrm{GG} \right]_{c}-k_{\mathrm{in}}\mathrm{CIF}\left[ \mathrm{GG} \right]_{c}$ | () |
| $\frac{d\left[ S2 \right]_{n}}{\mathrm{dt}}=k_{\mathrm{in}}\left[ S2 \right]_{c}-k_{\mathrm{ex}}\left[ S2 \right]_{n}+k_{\mathrm{dephos}}\left[ pS2 \right]_{n}\left[ \mathrm{PPase} \right]$ | () |
| $\frac{d\left[ G \right]_{n}}{\mathrm{dt}}=k_{\mathrm{in}}\left[ G \right]_{c}-k_{\mathrm{ex}}\left[ G \right]_{n}+k_{\mathrm{dephos}}\left[ \mathrm{pG} \right]_{n}\left[ \mathrm{PPase} \right]$ | () |
| $\frac{d\left[ pS2 \right]_{n}}{\mathrm{dt}}=k_{\mathrm{in}}\left[ pS2 \right]_{c}-k_{\mathrm{ex}}\left[ pS2 \right]_{n}-k_{\mathrm{dephos}}\left[ pS2 \right]_{n}\left[ \mathrm{PPase} \right] -k_{\mathrm{on}}\left[ pS2 \right]_{n}\left( \left[ S4 \right]_{n}+2\left[ pS2 \right]_{n}+\left[ \mathrm{pG} \right]_{n} \right)+k_{\mathrm{off}}\left( \left[ S24 \right]_{c}+2\left[ S22 \right]_{c}+\left[ G2 \right]_{c} \right)$ | () |
| $\frac{d\left[ \mathrm{pG} \right]_{n}}{\mathrm{dt}}=k_{\mathrm{in}}\left[ \mathrm{pG} \right]_{c}-k_{\mathrm{ex}}\left[ \mathrm{pG} \right]_{n}-k_{\mathrm{dephos}}\left[ \mathrm{pG} \right]_{n}\left[ \mathrm{PPase} \right] -k_{\mathrm{on}}\left[ \mathrm{pG} \right]_{n}\left( \left[ S4 \right]_{n}+\left[ pS2 \right]_{n}+{2\left[ \mathrm{pG} \right]}_{n} \right)+k_{\mathrm{off}}\left( \left[ G4 \right]_{c}+2\left[ G2 \right]_{c}+\left[ \mathrm{GG} \right]_{c} \right)$ | () |
| $\frac{d\left[ S4 \right]_{n}}{\mathrm{dt}}=k_{\mathrm{in}}\left[ S4 \right]_{c}-k_{\mathrm{in}}\left[ S4 \right]_{n}-k_{\mathrm{on}}\left[ S4 \right]_{n}\left( \left[ pS2 \right]_{n}+\left[ \mathrm{pG} \right]_{n} \right)+k_{\mathrm{off}}\left( \left[ S24 \right]_{n}+\left[ G4 \right]_{n} \right)$ | () |
| $\frac{d\left[ S24 \right]_{n}}{\mathrm{dt}}=k_{\mathrm{on}}\left[ pS2 \right]_{n}\left[ S4 \right]_{n}-k_{\mathrm{off}}\left[ S24 \right]_{n}+k_{\mathrm{in}}\mathrm{CIF}\left[ S24 \right]_{c}$ | () |
| $\frac{d\left[ G4 \right]_{n}}{\mathrm{dt}}=k_{\mathrm{on}}\left[ \mathrm{pG} \right]_{n}\left[ S4 \right]_{n}-k_{\mathrm{off}}\left[ G4 \right]_{n}+k_{\mathrm{in}}\mathrm{CIF}\left[ G4 \right]_{c}$ | () |
| $\frac{d\left[ S22 \right]_{n}}{\mathrm{dt}}=k_{\mathrm{on}}\left[ pS2 \right]_{n}^{2}-k_{\mathrm{off}}\left[ S22 \right]_{n}+k_{\mathrm{in}}\mathrm{CIF}\left[ S22 \right]_{c}$ | () |
| $\frac{d\left[ G2 \right]_{n}}{\mathrm{dt}}=k_{\mathrm{on}}\left[ \mathrm{pG} \right]_{n}\left[ pS2 \right]_{n}-k_{\mathrm{off}}\left[ G2 \right]_{n}+k_{\mathrm{in}}\mathrm{CIF}\left[ G2 \right]_{c}$ | () |
| $\frac{d\left[ \mathrm{GG} \right]_{n}}{\mathrm{dt}}=k_{\mathrm{on}}\left[ \mathrm{pG} \right]_{n}^{2}-k_{\mathrm{off}}\left[ \mathrm{GG} \right]_{n}+k_{\mathrm{in}}\mathrm{CIF}\left[ \mathrm{GG} \right]_{c}$ | () |

where, subscripts $n$ and $c$ represent nucleus and cytoplasm, R represents the unbound receptor, superscripts $act$ and $inact$ represent TGF-β activated receptor and SB (inhibitor) bound receptor, SB is the inhibitor, S2 is Smad2, pS2 is Phospho-Smad2, S4 is Smad4, S24 is Smad2/Smad4 complex, S22 is Samd2/Smad2 complex, PPase is Phosphatase, G is EGFP-Smad2, pG is the phosphorylated EGFP-Smad2, G4 is the EGFP-Smad2/Smad4 complex, G2 is EGFP-Smad2/Smad2 complex, and GG is the EGFP-Smad2/EGFP-Smad2 complex.

The parameter $k$ stands for rate constants in the mathematical model. The subscripts denote the process that the two reacting species are involved in. $k_{TGF-}$denotes the activation of receptors in the presence of TGF-β. The parameters $k_{on}$ and $k_{off}$ denote binding and unbinding rates for Phospho-Smad2 and Smad4, while $k_{onSB}$ and $k_{offSB}$ denote binding and unbinding rates for activated receptor ($R^{act}$) and the inhibitor (SB). The parameters $k_{in}$ and $k_{out}$ denote the rate at which complexes translocate in and out of the nucleus and, $k_{dephos}$ denotes the dephosphorylation rate of Phospho-Smad2 by Phosphatase. CIF is the complex import factor, which is the fold difference between the import rate of Smad complexes and import rate of monomeric Smads.

### Simulation conditions

We extracted data with error bars from Fig 2D in Schmierer et al. (2008), to obtain the dynamic nuclear EGFP-Smad2 concentration (Schmierer et al. 2008). We modeled the system of equations in two parts: (1) 𝑇GF-β induces pathway activation at t = 0, (2) an inhibitor, turns off the signaling cascade is added at t = 45 min. For part (1), we simulated the model equations for 45 mins with no inhibitor. For part (2), we added an inhibitor to the system and simulated with concentration at $t$ = 45 min as the initial conditions. We simulated this system of equations using ode15s in MATLAB. The objective function for this model is the sum of squared errors between the modeled profile and nuclear EGFP-Smad2 concentrations reported in Fig 2D by Schmierer et al. (2008), normalized by the error bars reported in Fig 2D from Schmierer et al. (2008). The initial concentrations of the species reported are as in the table below. The rest of the Smad species have a zero initial concentration.

| Species | Nucleus (nM) | Cytoplasm (nM) |
| --- | --- | --- |
| Smad 2 + EGFP-Smad2 | 28.5 + 28.5 | 60.6 + 60.6 |
| Smad4 | 50.8 | 50.8 |
| Other Smads | 0 | 0 |
| Inactive receptors | 1 |  |
| Phosphatase |  | 1 |
| 𝑇GF-β |  | 0.066 |
| SB (inhibitor) |  | 10,000 |

### Model results

In the Fig. S3, we plot the concentration of the nuclear EGFP-Smad2 complex data against the predicted value by one a high fit model parameter set. Please refer to Fig 2D in the Schmierer et al. (2008) for comparison.

| 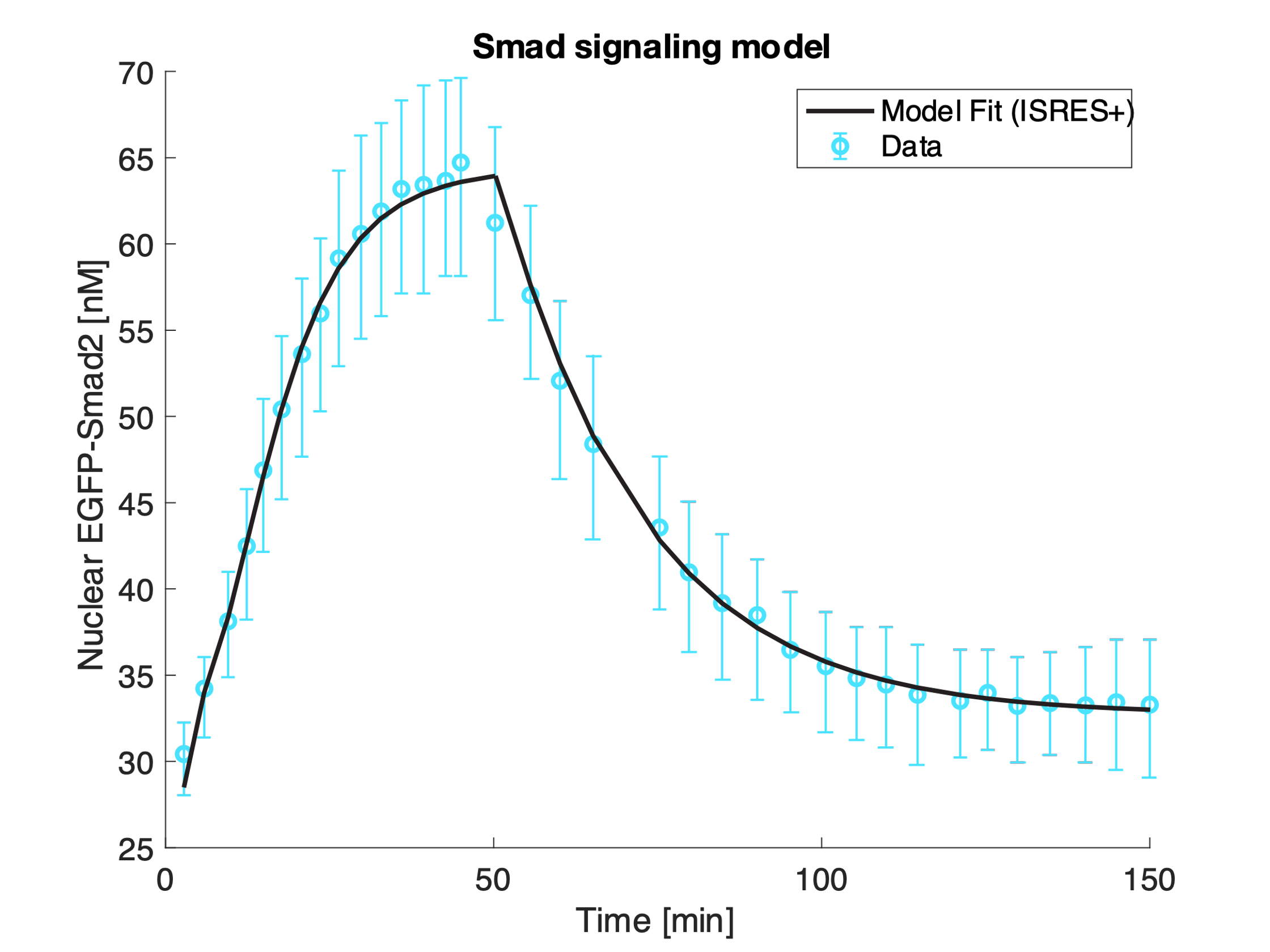  **Fig S3. Model and experimental concentration profile for EGFP-Smad2.** The plots represent the concentration profiles of EGFP-Smad2 with the addition of TGF-β at t = 0 min and after the addition of the inhibitor at t = 45 min. |
| --- |

## Gap gene circuit model

### Description

In *Drosophila* development*,* the positions of the 14 body segments in the body of an adult fly are determined to a high degree of precision within ~3hr of development after egg lay (Jäckle et al. 1992). This is accomplished by the Bicoid (Bcd) morphogen that is localized near the anterior pole of the embryo and during early development, it is expressed as a gradient along the anterior-posterior (A-P) direction (Driever and Nusslein-Volhard 1988). Bcd initiates an asymmetric gene expression cascade in the A-P direction by first activating gap genes that are expressed in broad overlapping domains, followed by pair-rule genes that are expressed in the seven stripes that determine the location of the 14 body segments In this model, the regulatory interactions between the gene regulatory network (GRN) components of the genes involved up to the gap gene part of the cascade are modeled (Jaeger et al. 2004; Manu et al. 2009).

### Mathematical model

The gap gene circuit model denotes the intracellular dynamics of protein concentrations along the nuclei in the 35-92% anterior-posterior position during nuclear cycle (nc) 13 and nc 14. This model for gap gene circuit includes Bcd, Cad, Hb, Kr, Kni, Gt, and the terminal gap gene Tll (Manu et al. 2009; Surkova et al. 2009) .

The mathematical model is expressed as follows,

| $\frac{d\nu_{i}^{a}}{dt}=R^{a}g\left( \sum_{b=1}^{N} T^{ab}\nu_{i}^{a}+m^{a}\nu_{i}^{Bcd}+\sum_{\beta=1}^{N_{e}} E^{a\beta}\nu_{i}^{\beta}\left( t \right)+h^{a} \right)+D^{a}\left[ \left( \nu_{i-1}^{a}-\nu_{i}^{a} \right)+\left( \nu_{i+1}^{a}-\nu_{i}^{a} \right) \right]-\lambda^{a}\nu_{i}^{a}$ | (1) |
| --- | --- |

where superscripts $a, b\in\left( 1,\ldots, N \right)$; N is the number of proteins, namely Hb, Kr, Kni, Gt; superscripts $\beta\in\left( 1,\ldots, N_{e} \right)$; $N_{e}$ is the number of time-varying inputs (Cad and Tll); subscript $i$ $\in(1,\ldots, M)$; and M is the number of nuclei. The three terms on the right side of the equation represent protein synthesis, Fickian diffusion and first order protein degradation.

Here,

$$g\left( u \right)=\frac{1}{2}\left[ \left( \frac{u}{\sqrt{u^{2}+1}} \right)+1 \right]$$

$g$is the sigmoidal regulation function, $\nu_{i}^{a}\left( t \right)$ is the concentration of the $a^{th}$ species in the $i^{th}$ nucleus at time t, $R^{a}$ is the maximum synthesis rate of species $a$, $T^{ab}$ and $E^{\alpha\beta}$ are coefficients that weight the transcriptional effect of species b or species β on species a (positive $\Rightarrow$ activation, negative $\Rightarrow$ repression), and $h_{a}$ represents the effect of ubiquitous transcription factors and sets threshold for activation.

### Simulation Conditions

The four gap genes; Hb, Kr, Kni, Gt; are expressed along the A-P axis from 35% AP position through 92% AP position. During nc 13, there are 40 nuclei in this region of the AP axis, and during nc 14, there are 57 nuclei. We discretized in space by the number of nuclei, so that there are 40 and 57 1D points in ncs 13 and 14, respectively, resulting in 160 and 228 coupled ordinary differential equations (ODEs) for ncs 13 and 14, respectively. For nc 13, ode15s integrates over the ODEs using the initial conditions of Hb, Kr, Kni, Gt expression (zeros for Kr, Kni, Gt, and experimentally derived for Hb). Sixteen minutes after the start of nc 13, the embryo enters mitosis, during which time the activation function, $g(u)$, is set to zero, but the remaining terms on the right-hand side of Eq (1) remain active. At time $t=21.1$ min after the start of nc 13, the embryo enters the interphase of nc 14, and the values of the concentrations of the four gap genes and three maternal proteins (Bcd, Cad, and Tll) from the end of nc 13 mitosis are smoothly interpolated onto a new spatial mesh of 57 points, which are then used as initial conditions for nc 14. The activation function, $g(u)$, is no longer set to zero during nc 14 interphase.

### Model Results

| **Fig S2. Model and experimental data for the concentration profiles of the four gap genes**. The plots represent the concentration profiles of Kr, Gt, Hb, and Kni along the anterior-posterior axis based on the conditions specified by the gap gene circuit model. 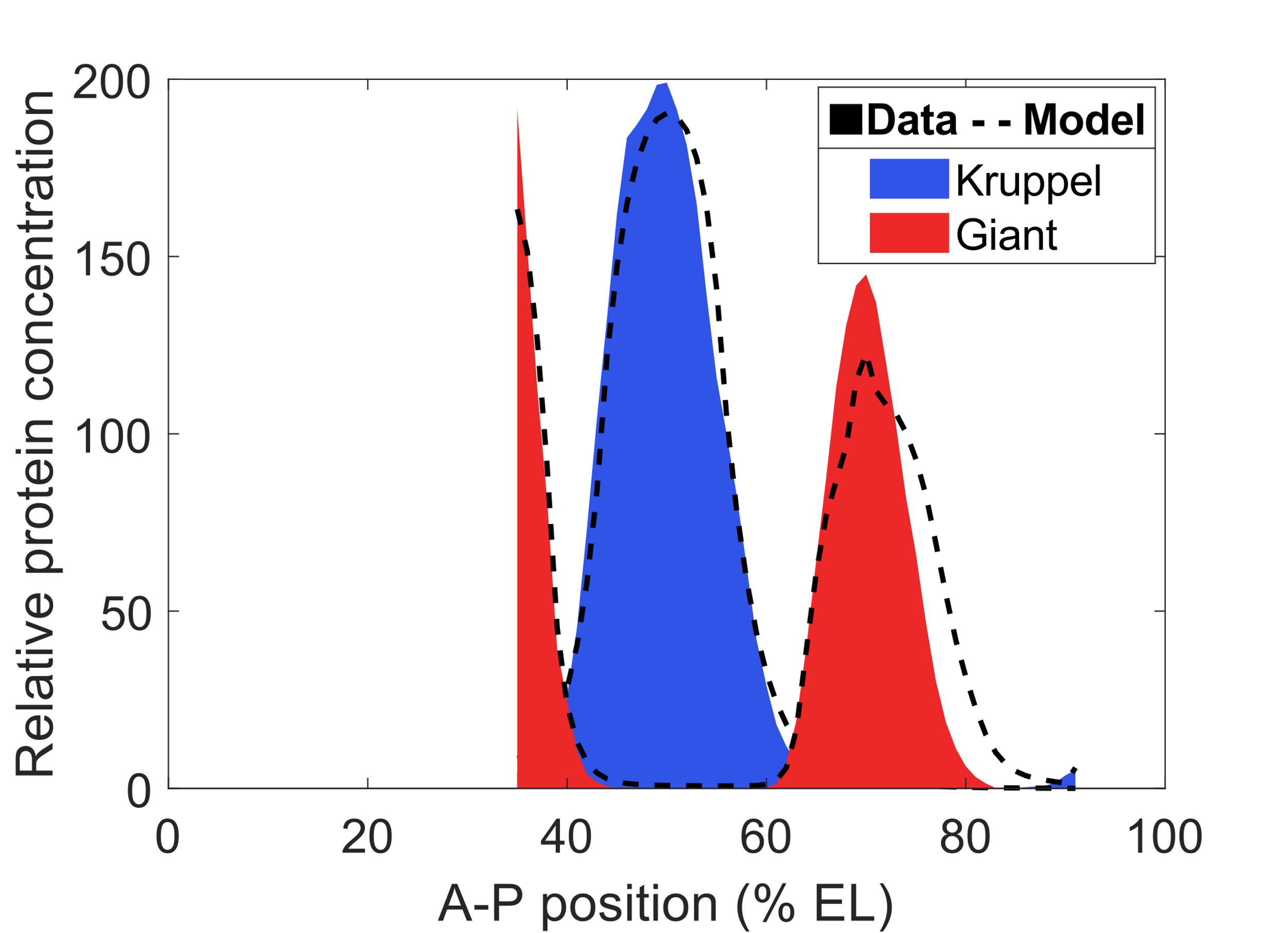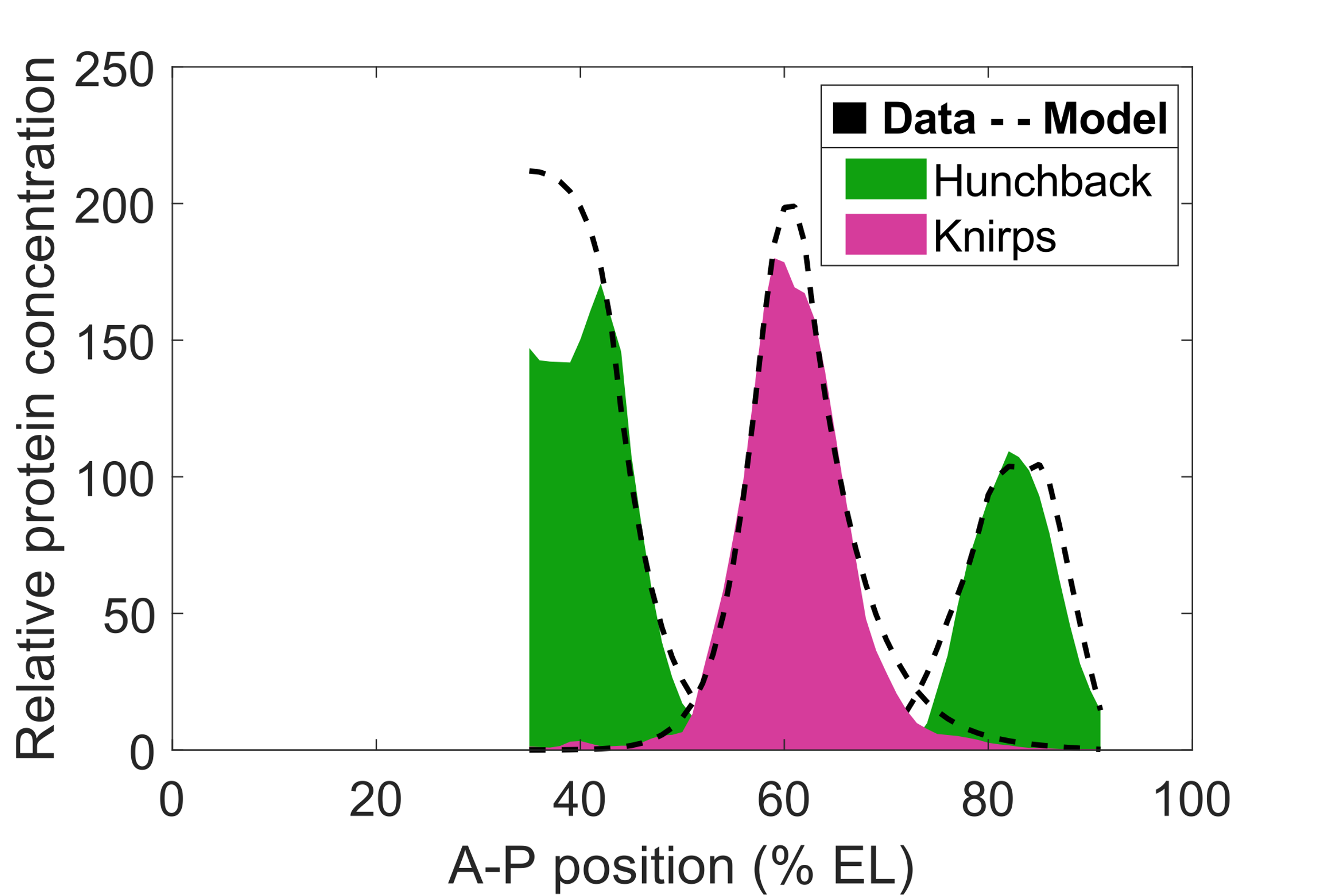 |
| --- |

In Fig. S2, we plot the expression profiles of the four gap genes, namely, Hb, Kr, Gt, and Kni*,* against model predictions from one of the highly fit (low error) parameter set.

# Derivation of Linstep and Newton step

## Linstep

The Linstep fitting equation may be represented as follows,

$$\boldsymbol{f}_{\boldsymbol{i}}=a_{0}+a_{1}\boldsymbol{\theta}_{\boldsymbol{1}}^{\boldsymbol{i}}+a_{2}\boldsymbol{\theta}_{\boldsymbol{2}}^{\boldsymbol{i}}+ .. +a_{n}\boldsymbol{\theta}_{\boldsymbol{n}}^{\boldsymbol{i}}$$

$$\boldsymbol{f}_{\boldsymbol{i}}= a_{0}+ \sum_{j=1}^{n} a_{j}\theta_{j}^{i}$$

where, $i=1 .. N$ are the number of individuals used in the fitting procedure.

$$\left[ \begin{matrix} f_{1} \\ \begin{matrix} f_{2} \\ \begin{matrix} \vdots\\ f_{N} \end{matrix} \end{matrix} \end{matrix} \right]=\left[ \begin{matrix} \begin{matrix} 1 & \theta_{1}^{1} & \theta_{2}^{1} \end{matrix} & \ldots& \theta_{n}^{1} \\ \begin{matrix} 1 & \theta_{1}^{2} & \theta_{1}^{1} \end{matrix} & \ldots& \theta_{n}^{2} \\ \begin{matrix} \begin{matrix} 1 \\ \vdots\\ 1 \end{matrix} & \begin{matrix} \theta_{1}^{3} \\ \vdots\\ \theta_{1}^{N} \end{matrix} & \begin{matrix} \theta_{1}^{1} \\ \vdots\\ \theta_{1}^{N} \end{matrix} \end{matrix} & \begin{matrix} \ldots\\ \vdots\\ \ldots\end{matrix} & \begin{matrix} \theta_{n}^{3} \\ \vdots\\ \theta_{n}^{N} \end{matrix} \end{matrix} \right] \left[ \begin{matrix} a_{0} \\ \begin{matrix} a_{1} \\ \vdots\\ a_{N} \end{matrix} \end{matrix} \right]$$

$$a= \left( M^{T}M^{-1} \right)M^{T}F$$

## Newton step

The Newton step fitting equation may be represented as follows,

$$\boldsymbol{f}\left( \boldsymbol{\theta}^{i} \right)= \boldsymbol{f}\left( \boldsymbol{\theta}^{\boldsymbol{^{\circ}}} \right)+\boldsymbol{\nabla}\boldsymbol{f}_{\boldsymbol{\theta}^{\boldsymbol{^{\circ}}}} \Delta\boldsymbol{\theta}+ \frac{1}{2}\Delta\boldsymbol{\theta}^{T}\mathbf{H}_{\mathbf{0}}\Delta\boldsymbol{\theta}$$

where $\Delta\boldsymbol{\theta}=\boldsymbol{\theta}^{i}-\boldsymbol{\theta}^{\boldsymbol{^{\circ}}}$, where $\boldsymbol{\theta}^{\boldsymbol{^{\circ}}}$ and $\boldsymbol{\theta}^{\boldsymbol{i}}$ refers to the fittest individual in the population and to individual $i$, and $\mathbf{H}_{\boldsymbol{0}}$ is the Hessian at the $\boldsymbol{\theta=}\boldsymbol{\theta}^{\boldsymbol{^{\circ}}}$.

The third term can be written as,

$$\frac{1}{2}\Delta\boldsymbol{\theta}^{T}\mathbf{H}_{\mathbf{0}}\Delta\boldsymbol{\theta=}\left[ \begin{matrix} \Delta\theta_{1} & \Delta\theta_{2} & \begin{matrix} \cdots& \Delta\theta_{n} \end{matrix} \end{matrix} \right]\left[ \begin{matrix} \begin{matrix} H_{11} & H_{12} & H_{13} \end{matrix} & \ldots& H_{1n} \\ \begin{matrix} H_{21} & H_{22} & H_{23} \end{matrix} & \ldots& H_{2n} \\ \begin{matrix} \begin{matrix} H_{31} \\ \vdots\\ H_{n1} \end{matrix} & \begin{matrix} H_{32} \\ \vdots\\ H_{n2} \end{matrix} & \begin{matrix} H_{33} \\ \vdots\\ H_{n3} \end{matrix} \end{matrix} & \begin{matrix} \ldots\\ \vdots\\ \ldots\end{matrix} & \begin{matrix} H_{3n} \\ \vdots\\ H_{nn} \end{matrix} \end{matrix} \right] \left[ \begin{matrix} \Delta\theta_{1} \\ \begin{matrix} \Delta\theta_{2} \\ \vdots\\ \Delta\theta_{n} \end{matrix} \end{matrix} \right]$$

$$= \frac{1}{2}\sum_{i=1}^{n} \sum_{j=1}^{n} H_{jk}\Delta\theta_{j}\Delta\theta_{k}$$

where $H_{jk}= \frac{\partial^{2}f}{\partial\theta_{j}\partial\theta_{k}}$ and $\Delta\theta_{k}=\theta_{k}^{i}-\theta_{k}^{^{\circ}}$. The term can be simplified as follows,

$$\frac{1}{2}\sum_{i=1}^{n} \sum_{j=1}^{n} H_{jk}\Delta\theta_{j}\Delta\theta_{k}= \frac{1}{2}\sum_{i=1}^{n} \sum_{j=1}^{n} H_{jk}\left( \theta_{j}^{i}- \theta_{j}^{^{\circ}} \right)\left( \theta_{k}^{i}- \theta_{k}^{^{\circ}} \right)$$

$$= \frac{1}{2}\sum_{i=1}^{n} \sum_{j=1}^{n} H_{jk}\left( \theta_{j}^{i}\theta_{k}^{i}- \theta_{j}^{i}\theta_{k}^{^{\circ}}- \theta_{j}^{^{\circ}}\theta_{k}^{i}+ \theta_{j}^{^{\circ}}\theta_{k}^{^{\circ}} \right)$$

$$= \frac{1}{2}\sum_{i=1}^{n} \sum_{j=1}^{n} \left( H_{jk}\theta_{j}^{i}\theta_{k}^{i}+ {a_{jk}\theta}_{j}^{i}+ b_{jk}\theta_{j}^{^{\circ}}\theta_{k}^{i}+ c_{jk} \right)$$

where $a_{jk}= -H_{jk}\theta_{k}^{^{\circ}}$, $b_{jk}= -H_{jk}\theta_{j}^{^{\circ}}$, and $c_{jk}= -H_{jk}\theta_{j}^{^{\circ}}\theta_{k}^{^{\circ}}$.

The second term may be written as,

$$\boldsymbol{\nabla}\boldsymbol{f} \Delta\boldsymbol{\theta=}\left[ \begin{matrix} f_{\theta_{1}} & f_{\theta_{2}} & \begin{matrix} \cdots& f_{\theta_{n}} \end{matrix} \end{matrix} \right]\left[ \begin{matrix} (\theta_{1}^{i}- \theta_{1}^{^{\circ}}) \\ \begin{matrix} (\theta_{2}^{i}- \theta_{2}^{^{\circ}}) \\ \vdots\\ (\theta_{n}^{i}- \theta_{n}^{^{\circ}}) \end{matrix} \end{matrix} \right]$$

$$= \sum_{j=1}^{n} f_{\theta_{j}}\left( \theta_{j}^{i}- \theta_{j}^{^{\circ}} \right)$$

Both the terms can be put together as follows,

$$\boldsymbol{f}_{\boldsymbol{i}}= \frac{1}{2}\sum_{j=1}^{n} \sum_{k=1}^{n} H_{jk}\theta_{j}^{i}\theta_{k}^{i}+ \sum_{j=1}^{n} g_{j}\theta_{j}^{i}+c$$

where $\boldsymbol{g}_{\boldsymbol{j}}=f_{\theta_{j}}- \sum_{k=1}^{n} H_{jk}\theta_{k}^{^{\circ}}$and $c= \sum_{j=1}^{n} f_{\theta_{j}^{^{\circ}}}- \sum_{j=1}^{n} f_{\theta_{j}}\theta_{j}^{^{\circ}}+ \frac{1}{2}\sum_{j=1}^{n} \sum_{k=1}^{n} H_{jk}\theta_{j}^{^{\circ}}\theta_{k}^{^{\circ}}.$ Thus, the elements of the Hessian matrix and the elements of the gradient can be estimated by inverting the matrix consisting of combinations of $\boldsymbol{\theta}^{\boldsymbol{i}}$ on the right hand side similar to Linstep.

## A full Newton step leads all individuals considered in the Hessian calculation to move to the same point

Differentiating the second order fit equation,

$$\boldsymbol{f}\left( \boldsymbol{\theta}^{i} \right)= \boldsymbol{f}\left( \boldsymbol{\theta}^{\boldsymbol{^{\circ}}} \right)+\boldsymbol{\nabla}\boldsymbol{f}_{\boldsymbol{\theta}^{\boldsymbol{^{\circ}}}} \Delta\boldsymbol{\theta}+ \frac{1}{2}\Delta\boldsymbol{\theta}^{T}\mathbf{H}_{\mathbf{0}}\Delta\boldsymbol{\theta}$$

with respect to $\theta$ gives,

$$0= \left( \boldsymbol{\nabla}\boldsymbol{f}^{\boldsymbol{^{\circ}}} \right)^{T}+\mathbf{H}^{\boldsymbol{^{\circ}}}\Delta\theta$$

Therefore,

$$\boldsymbol{\theta}^{\boldsymbol{i}}=\boldsymbol{\theta}^{\boldsymbol{^{\circ}}}- {(\mathbf{H}^{\boldsymbol{^{\circ}}}\boldsymbol{)}}^{-1}\left[ \left( \boldsymbol{\nabla}\boldsymbol{f}^{\boldsymbol{^{\circ}}} \right)^{T} \right]$$

Using the definition of $g_{j}$ from the above derivation, and rearranging give,

$$f_{\theta_{j}}=g_{j}+ \sum_{k=1}^{n} H_{jk}\theta_{k}^{^{\circ}}$$

we can rewrite the equation as,

$$\boldsymbol{\theta}^{\boldsymbol{i}}=\boldsymbol{\theta}^{\boldsymbol{^{\circ}}}- {(\mathbf{H}^{\boldsymbol{^{\circ}}}\boldsymbol{)}}^{-1}\left[ \boldsymbol{g}+ \mathbf{H}^{\boldsymbol{^{\circ}}}\theta^{^{\circ}} \right]$$

$$=\left( \mathbf{H}^{\boldsymbol{^{\circ}}} \right)^{-1}\boldsymbol{g}$$

# The gap gene circuit model with Linstep and Newton step active throughout

In Fig 4, we show that ISRES+ performs better than ISRES when Linstep is active in the early generations and Newton step is active in late generations. Systems biology models are complex in nature and have complex manifold structures, which makes it difficult to generalize the performance of ISRES+ when compared to ISRES. The configurations with which ISRES+ performs better than ISRES depends on a lot of factors (see Discussion), and the hyperparameters we used in Fig 4 are just recommendations. As a test of what is necessary, we compare the performance of ISRES and ISRES+ using Linstep active throughout, Newton step active throughout and both steps active throughout strategy. In the Linstep-only mode ISRES+ has a comparable performance to ISRES in the early generations (Fig S4A), but in the late generations even though the distribution of $\log_{10} (inverse fitness)$ is slightly tighter for ISRES, the distribution of inverse fitness of the fittest individual over all generations over all runs (N > 50) is not significantly different compared to ISRES (FIG S4-B). In Newton step only mode, ISRES+ performs marginally better than ISRES in the early generations but underperforms in the late generations (Fig S4 C). Overall, the final distribution of the inverse fitness of all the independent simulations is similar for ISRES+ and ISRES (Fig S4 D). With both Linstep and Newton step active, ISRES+ continues to underperform throughout (Fig S4 E) and has a significant number of fittest individuals with a higher inverse fitness (Fig S4 F).

In conclusion, for the gap gene circuit model, the three modes: Linstep only, Newton step only and both Linstep and Newton step which perform well for the other two models do not perform well, and ISRES+ has a comparable performance to ISRES.

| ****  **Fig S4: The gap gene circuit model with both Linstep and Newton step active throughout.** (A) The three plot lines represent 75^th^, 50^th^ and 25^th^ percentile of all the independent simulations (N>50). The plot indicates the $\log_{10} (inverse fitness)$across generations. (B) Histogram plot of the inverse fitness of the fittest individual over all generations from all independent simulations. (A-B) ISRES+ was run with only Linstep active for all 3000 generations. (A) ISRES and ISRES+ have comparable median performance in the first ~300 generations. ISRES+ catches on between 500-1500 generations but the final distribution is not significantly different from ISRES. (B) With only Linstep active for all 3000 generations ISRES+ finds a greater number of solutions with a better inverse fitness but the distribution of inverse fitness of the fittest individual over all generations over all runs (N > 50) is not significantly different compared to ISRES. (C-D) ISRES+ was run with only Newton step on for all 3000 generations. (C) ISRES+ and ISERS has comparable performance in the first 500 generations. The 50^th^ percentile plotline indicates that ISRES+ gets better than ISRES between 500 and 1500 generations but the performance of ISRES+ worsens after 1500 generations such that both ISRES+ and ISRES have a $\log_{10} (inverse fitness)$comparable distribution. (D) With an Newton step only configuration the final distribution of the inverse fitness of the fittest individual over all generations over all runs (N>50) is not significantly different compared to ISRES. (E-F) ISRES+ was run with Linstep and Newton step active throughout. (E) ISRES+ has a comparable performance to ISRES in the early generations and gets worse in the later generations. The final distribution of ISRES+ and ISRES are comparable, but ISRES performs better than ISRES+ at the median plot line. (F) With both Linstep and Newton step on throughout the final distribution of the inverse fitness of the fittest individual over all generations over all runs (N>50) is significantly better for ISRES than ISRES+. (*p* value was calculated using a modified t-test) |
| --- |

# The effect of varying β_lin_

As discussed before in the Algorithm and Methodology sections, unlike Newton step, Linstep does not have a built-in step size and its step size depends on the value of β_lin_. β_lin_ is the Linstep parameter that controls the magnitude of the gradient-descent step with respect to the diameter of the cluster bubble formed by the individuals used to determine the gradient. Values of this parameter greater than one indicate that the magnitude of the gradient descent step is larger than the diameter of the cluster bubble. While theoretically, any value of β_lin_ ≥ 1 would work, practically, a value not too much higher than one would be most appropriate to protect the solution from deviating too far away from the bubble into unchartered terrain.

For the Dorsal/Cactus model, ISRES+ has the best performance with a β_lin_ = 1. We tested several values of β_lin_ : 1, 1.5, 2 and 10 (Fig S5 A-L). ISRES+ performs significantly better than ISRES at β_lin_ = 1 (Fig S5 A-C) and at all other values of β_lin_, ISRES+ progressively loses its effectiveness on increasing β_lin_ to 10. At β_lin_ = 1.5, ISRES+ performs better than ISRES (but less significantly compared to β_lin_ = 1) and has a tighter distribution of inverse fitness (Fig S5 D-F). At β_lin_ = 2, ISRES+ and ISRES have a comparable performance (Fig S5 G-I), and at β_lin_ = 10, even though ISRES+ and ISRES have a comparable median performance, ISRES+ has a tighter final distribution of inverse fitness in the later generations (Fig S5 J-L).

For the Smad signaling model, there is a marginal difference in the performance of ISRES+ at β_lin_ values of 0.05, 0.1, 0.25, 0.5 and 0.75 (Fig S6 A-J). Therefore, at values of β_lin_ < 1, ISRES+ performs better than ISRES but there is very little difference in the performance of ISRES+ across β_lin_ values. Whereas at β_lin_ > 1, ISRES+ has a similar performance at β_lin_ = 1 and β_lin_ = 1.25 (Fig S6 K-N), but the performance increases by a lot at β_lin_ = 1.5 (Fig S6 O-P). For β_lin_ = 1.75, ISRES+ has a comparable performance to ISRES (Fig S6 Q-R) but as the β_lin_ value is increased to 2 (Fig S6 S-T), ISRES+ again performs better than ISRES. In summary, the effect of β_lin_ on the results is not directly correlated but certain values of β_lin_ the algorithm performs better than ISRES and at certain values it performs worse.

For the gap gene circuit model, we tested multiple values of β_lin_ (Fig S7) and found that β_lin_ = 2 (Fig 4 CD,EF) has the best performance. We systematically varied the value of β_lin_ from 1 to 10 and compared the performance of ISRES+ to ISRES. For β_lin_ = 1, ISRES performs significantly better than ISRES+ and has a better final distribution of the inverse fitness of the fittest individuals over all generations. The performance of ISRES+ at β_lin_ = 1.5 compared to ISRES was slightly better than ISRES, such that ISRES+ has a greater number of fitter individuals at lower inverse fitness than ISRES (Fig S7 D) but the 25^th^, 50^th^ and 75^th^ percentile plotlines almost overlap. As discussed in the Results section, ISRES+ with β_lin_ = 2 has the best performance, even though the median plotline indicates that ISRES+ underperforms in the early generations (Fig S7 E), in generations post-500 ISRES+ starts to perform better (Fig S7 E) and has a greater number of fitter individuals with a lower. Inverse fitness (Fig S7 F). For β_lin_ = 3, there is no significant difference between the percentile plots and the final distribution of inverse fitness of fittest individual of ISRES+ and ISRES (Fig S7 K-L). This trend continued as we increased β_lin_ to 5 and then 10 (Fig S7 M-P).

Since ISRES+ performs well with a β_lin_ = 2 but not with β_lin_ = 3, we also tested intermediate values of β_lin_ = 2.25 and 2.5 (Fig S7 G-K). ISRES+ loses its gains on going from 2 to 3 gradually. At β_lin_ = 2.25, ISRES+ has a higher number of individuals with a lower inverse fitness compared to ISRES (Fig S7 G-H), but the difference is less significant at β_lin_ = 2.5.

Note that for β_lin_ = 1.5, we ran both ISRES and ISRES+ for 5000 generations to test whether either algorithm converge to a better solution if ran for more generations. And, both ISRES+ and ISRES displayed a similar performance in the final distribution of fittest individuals over all generations when ran for 3000 or 5000 (Fig S7 CD) generations.

Also note that, for β_lin_ = 10 (Fig S7 KL), only Linstep is active.

In summary, with β_lin_ = 2 ISRES+ has the best performance for the gap gene circuit model whereas ISRES+ has a comparable performance to ISRES at all other values of β_lin_ we tested.

|   **Fig S5: The effect of varying β_lin_ for the Dl/Cact model.** (A) The three plot lines represent 25*^th^*, 50*^th^* and 75*^th^* percentile of all the independent simulations (N>50). The plot indicates the ${log}_{10} inverse fitness$ across generations. (B) The plot in (A) was zoomed-in to compare the plot lines in the last 500 generations (C) Histogram plot of the inverse fitness of the fittest individual over all generations from all independent simulations. (A-C) ISRES+ was run with Linstep (β_lin_ = 1) and Newton step active throughout. (A-B) ISRES+ has a comparable performance to ISRES at the 25^th,^ 50^th^ and the 75^th^ percentile line in the early generations. But in the late generations ISRES+ has a lower inverse fitness at all three plot lines and has a tighter distribution compared to ISRES. (C) Histogram plot of the inverse fitness of the fittest individual over all generations from all independent simulations indicates that ISRES+ has significantly more data points in the lower inverse fitness region compared to ISRES. (D-F) ISRES+ was run with Linstep (β_lin_ = 1.5) and Newton step active throughout. (D-E) ISRES+ has a comparable performance to ISRES at the 25^th,^ 50^th^ and the 75^th^ percentile line in the early generations. But in the late generations ISRES+ has a lower inverse fitness at all three plot lines and has a tighter distribution compared to ISRES. (F) Histogram plot of the inverse fitness of the fittest individual over all generations from all independent simulations indicates that ISRES+ has significantly more data points in the lower inverse fitness region compared to ISRES. (G-I) ISRES+ was run with Linstep (β_lin_ = 2) and Newton step active throughout. (G-H) ISRES+ has a comparable performance to ISRES at the 25^th,^ 50^th^ and the 75^th^ percentile line in the early generations and late generations (I) Histogram plot of the inverse fitness of the fittest individual over all generations from all independent simulations indicates there is no significant difference between ISRES+ and ISRES. (J-L) ISRES+ was run with Linstep (β_lin_ = 5) and Newton step active throughout. (J-K) ISRES+ has a comparable performance to ISRES at the 25^th,^ 50^th^ and the 75^th^ percentile line in the early generations and later generations, but ISRES+ has a tighter distribution of inverse fitness. (L) Histogram plot of the inverse fitness of the fittest individual over all generations from all independent simulations indicates there is no significant difference between ISRES+ and ISRES. (*p* value was calculated using Wilcoxon test in MATLAB) |
| --- |

|   **Fig S6: The effect of varying β_lin_ for the Smad signaling model.** (A) ISRES+ was run with both Linstep (β_lin_ = 0.05) and Newton step active throughout. ISRES+ finds the lower minima in ~75% of the runs, as opposed to ~60% for ISRES. (B) ISRES+ was run with both Linstep (β_lin_ = 0.1) and Newton step on throughout. ISRES+ finds the lower minima in ~80% of the runs, as opposed to ~60% for ISRES. (C) ISRES+ was run with both Linstep (β_lin_ = 0.25) and Newton step on throughout. ISRES+ finds the lower minima in ~80% of the runs, as opposed to ~60% for ISRES. (D) ISRES+ was run with both Linstep (β_lin_ = 0.5) and Newton step on throughout. ISRES+ finds the lower minima in ~70% of the runs, as opposed to ~60% for ISRES. (E) ISRES+ was run with both Linstep (β_lin_ = 0.75) and Newton step on throughout. ISRES+ finds the lower minima in ~80% of the runs, as opposed to ~60% for ISRES. (F) ISRES+ was run with both Linstep (β_lin_ = 1) and Newton step on throughout. ISRES+ finds the lower minima in ~88% of the runs, as opposed to ~60% for ISRES. (G) ISRES+ was run with both Linstep (β_lin_ = 1.25) and Newton step on throughout. ISRES+ finds the lower minima in ~80% of the runs, as opposed to ~60% for ISRES. (H) ISRES+ was run with both Linstep (β_lin_ = 1.50) and Newton step on throughout. ISRES+ finds the lower minima in >95% of the runs, as opposed to 60% for ISRES. (I) ISRES+ was run with both Linstep (β_lin_ = 1.75) and Newton step active throughout. ISRES+ finds the lower minima in ~70% of the runs, as opposed to ~60% for ISRES. (J) ISRES+ was run with both Linstep (β_lin_ = 2) and Newton step on throughout. ISRES+ finds the lower minima in ~84% of the runs, as opposed to ~60% for ISRES. |
| --- |

|  |
| --- |
| **Fig S7: The effect of varying β_lin_ for the gap gene circuit model.** (A) The three plot lines represent 25^th^, 50^th^ and 75^th^ percentile of all the independent simulations (N>50). The plot indicates the $\log_{10} (inverse fitness)$across generations. (B) Histogram plot of the inverse fitness of the fittest individual over all generations from all independent simulations. (A-B) ISRES+ (N = 76) was run with Linstep (β_lin_ = 1) and Newton step active throughout. (A) ISRES+ underperforms compared to ISRES at the 25^th^, 50^th^ and 75^th^ plot lines in the early generations. In the mid-generations ISRES+ has a comparable performance to ISRES and in the late generations ISRES performs slightly (and significantly) better that ISRES+. (B) The final distribution of inverse fitness of the fittest individual over all generations is significantly better for ISRES than ISRES+. (C-D) ISRES+ (N = 46) was run for 5000 generations with Linstep (β_lin_ = 1.5) and Newton step active throughout. (C) Throughout ISRES+ and ISRES have a comparable performance, with a marginal difference in the 25^th^ and 75^th^ percentile plot line between ISRES+ and ISRES, where ISRES performs better. (D) The final distribution of inverse fitness of the fittest individual over all generations is marginally better for ISRES than ISRES+. (E-F) ISRES+ (N = 99) was run with Linstep (β_lin_ = 2) and Newton step active throughout. (E) In the early generations, there is no significant difference between ISRES+ and ISRES, whereas in the later generations ISRES+ outperforms ISRES. (F) The final distribution of inverse fitness of the fittest individual over all generations is significantly better for ISRES than ISRES+, such that ISRES+ results in a greater number of individuals with a lower inverse fitness. (G-H) ISRES+ (N = 77) was run with Linstep (β_lin_ = 2.25) and Newton step active throughout. (G) In the early generations, there is no significant difference between ISRES+ and ISRES, whereas in the later generations ISRES+ outperforms ISRES at the 50^th^ percentile line. (H) The final distribution of inverse fitness of the fittest individual over all generations is better for ISRES than ISRES+, such that ISRES+ results in a greater number of individuals with a lower inverse fitness. (I-J) ISRES+ (N = 48) was run with Linstep (β_lin_ = 2.5) and Newton step active throughout. (I) ISRES+ and ISRES have a comparable performance. (J) The final distribution of inverse fitness of the fittest individual over all generations is slightly better for ISRES than ISRES+, such that ISRES+ results in slightly higher number of individuals with a lower inverse fitness. (K-L) ISRES+ (N = 52) was run with Linstep (β_lin_ = 3) and Newton step active throughout. (K) ISRES+ and ISRES have a comparable performance at the 25^th,^ 50^th^ and 75^th^ percentile plot line. (L) There is no significant difference between the final distribution of inverse fitness of the fittest individual over all generations for ISRES and ISRES+. (M-N) ISRES+ (N = 95) was run with Linstep (β_lin_ = 5) and Newton step active throughout. (M) ISRES+ and ISRES have a comparable performance at the 25^th,^ 50^th^ and 75^th^ percentile plot line. (N) There is no significant difference between the final distribution of inverse fitness of the fittest individual over all generations for ISRES and ISRES+. (O-P) ISRES+ (N = 95) was run with only Linstep (β_lin_ = 10) active throughout. (O) ISRES+ and ISRES have a comparable performance at the 25^th,^ 50^th^ and 75^th^ percentile plot line. (P) There is no significant difference between the final distribution of inverse fitness of the fittest individual over all generations for ISRES and ISRES+. (*p* value was calculated using a modified t-test) |

# Comparison of wall-clock times of ISRES and ISRES+

Linstep and Newton step require an approximate gradient and an approximate hessian to be computed to generate new individuals (see Methodology and Discussion). A linear model is used to build an estimate of the gradient, which is used to perform an approximate gradient descent step and a quadratic function model is used to build an estimate of the Hessian, which is used to perform an approximate Newton’s method of function optimization step. The overhead required to calculate this is minimal since neither the gradient nor the Hessian is computed. As shown in Fig S8, for all the three models the cumulative distribution frequency of wall-clock time for all independent runs of ISRES and ISRES+ are comparable. The Dorsal/Cactus (Dl/Cact) takes $O\left( t_{wall clock} \right)\approx100 \min$ to run for 1000 generations for both ISRES and ISRES+ (Fig S8A). The Smad signaling model takes $O\left( t_{wall clock} \right)\approx10 \min$ to run for 500 generations for both ISRES and ISRES+ (Fig S8B). The gap gene circuit model $O\left( t_{wall clock} \right)\approx10 h$ to run for 3000 generations for both ISRES and ISRES+ (Fig S8C). In general, for all three models presented in this paper, ISRES and ISRES+ both have a similar wall clock time, which means the calculation of Linstep and Newton step does not increase average run times.

|   **Fig S8: Comparing wall-clock time ISRES+ and ISRES for all three models.** (A) For the Dorsal/Cactus (Dl/Cact) model the cumulative distribution frequency of wall-clock time for all ISRES (N = 102) and ISRES+ (N = 52) runs almost overlap. (B) For the Smad signaling model the cumulative distribution frequency of wall-clock time for all ISRES (N = 137) and ISRES+ (N = 263) runs are comparable. (C) For the gap gene circuit model, (note that the time duration is in hours) ISRES (N = 108) and ISRES+ (N = 311) have a comparable cumulative distribution frequency of wall-clock time. |
| --- |

# References

Aashaq, Sabreena, Asiya Batool, Shabir Ahmad Mir, Mushtaq Ahmad Beigh, Khurshid Iqbal Andrabi, and Zaffar Amin Shah. 2022. "TGF‐β signaling: A recap of SMAD‐independent and SMAD‐dependent pathways." *Journal of Cellular Physiology* 237 (1):59-85. doi: 10.1002/jcp.30529.

Al Asafen, Hadel, Prasad U. Bandodkar, Sophia Carrell-Noel, Allison E. Schloop, Jeramey Friedman, and Gregory T. Reeves. 2020. "Robustness of the Dorsal morphogen gradient with respect to morphogen dosage." *PLOS Computational Biology* 16 (4):e1007750. doi: 10.1371/journal.pcbi.1007750.

Carrell, Sophia N., Michael D. O'Connell, Thomas Jacobsen, Amy E. Allen, Stephanie M. Smith, and Gregory T. Reeves. 2017. "A facilitated diffusion mechanism establishes the Drosophila Dorsal gradient." *Development* 144 (23):4450-4461. doi: 10.1242/dev.155549.

Driever, W., and C. Nusslein-Volhard. 1988. "A gradient of bicoid protein in Drosophila embryos." *Cell* 54 (1):83-93. doi: 10.1016/0092-8674(88)90182-1.

Huang, Shuan S., and Jung S. Huang. 2005. "TGF-β control of cell proliferation." *Journal of Cellular Biochemistry* 96 (3):447-462. doi: 10.1002/jcb.20558.

Jaeger, J., M. Blagov, D. Kosman, K. N. Kozlov, Manu, E. Myasnikova, S. Surkova, C. E. Vanario-Alonso, M. Samsonova, D. H. Sharp, and J. Reinitz. 2004. "Dynamical analysis of regulatory interactions in the gap gene system of Drosophila melanogaster." *Genetics* 167 (4):1721-1737. doi: 10.1534/genetics.104.027334.

Jäckle, Herbert, Michael Hoch, Michael J. Pankratz, Nicole Gerwin, Frank Sauer, and Günter Brönner. 1992. "Transcriptional control by <i>Drosophila</i> gap genes." *Journal of Cell Science* 1992 (Supplement_16):39-51. doi: 10.1242/jcs.1992.supplement_16.6.

Kanodia, Jitendra S., Richa Rikhy, Yoosik Kim, Viktor K. Lund, Robert Delotto, Jennifer Lippincott-Schwartz, and Stanislav Y. Shvartsman. 2009. "Dynamics of the Dorsal morphogen gradient." *Proceedings of the National Academy of Sciences* 106 (51):21707-21712. doi: 10.1073/pnas.0912395106.

Kitisin, Krit, Tapas Saha, Tiffany Blake, Nady Golestaneh, Merlyn Deng, Christine Kim, Yi Tang, Kirti Shetty, Bibhuti Mishra, and Lopa Mishra. 2007. "TGF-β signaling in development." *Science's STKE* 2007 (399):cm1-cm1.

Manu, S. Surkova, A. V. Spirov, V. V. Gursky, H. Janssens, A. R. Kim, O. Radulescu, C. E. Vanario-Alonso, D. H. Sharp, M. Samsonova, and J. Reinitz. 2009. "Canalization of Gene Expression in the Drosophila Blastoderm by Gap Gene Cross Regulation." *Plos Biology* 7 (3):591-603. doi: 10.1371/journal.pbio.1000049.

Massagué, Joan. 2012. "TGFβ signalling in context." *Nature Reviews Molecular Cell Biology* 13 (10):616-630. doi: 10.1038/nrm3434.

O’Connell, Michael D., and Gregory T. Reeves. 2015. "The Presence of Nuclear Cactus in the Early Drosophila Embryo May Extend the Dynamic Range of the Dorsal Gradient." *PLOS Computational Biology* 11 (4):e1004159. doi: 10.1371/journal.pcbi.1004159.

Schmierer, B., and C. S. Hill. 2007. "TGF beta-SMAD signal transduction: molecular specificity and functional flexibility." *Nature Reviews Molecular Cell Biology* 8 (12):970-982. doi: 10.1038/nrm2297.

Schmierer, B., A. L. Tournier, P. A. Bates, and C. S. Hill. 2008. "Mathematical modeling identifies Smad nucleocytoplasmic shuttling as a dynamic signal-interpreting system." *Proceedings of the National Academy of Sciences of the United States of America* 105 (18):6608-6613. doi: 10.1073/pnas.0710134105.

Schuster, Norbert, and Kerstin Krieglstein. 2002. "Mechanisms of TGF-β-mediated apoptosis." *Cell and Tissue Research* 307 (1):1-14. doi: 10.1007/s00441-001-0479-6.

Surkova, Svetlana, Alexander V Spirov, Vitaly V Gursky, Hilde Janssens, Ah-Ram Kim, Ovidiu Radulescu, Carlos E Vanario-Alonso, David H Sharp, Maria Samsonova, and John Reinitz. 2009. "Canalization of gene expression and domain shifts in the Drosophila blastoderm by dynamical attractors." *PLoS computational biology* 5 (3):e1000303.
